# Exhausted CD4^+^T cells are associated with CCL4-driven immunosuppressive macrophage accumulation in enzootic bovine leukosis

**DOI:** 10.3389/fimmu.2026.1808815

**Published:** 2026-05-04

**Authors:** Hayato Nakamura, Tomohiro Okagawa, Malik Hamaïdia, Wisa Tiyamanee, Mari Ikehata, Minagi Hirose, Maho Inoue, Luc Willems, Keisuke Maezono, Passawat Thammahakin, Shintaro Kobayashi, Yukinari Kato, Yasuhiko Suzuki, Naoya Maekawa, Shiro Murata, Kazuhiko Ohashi, Satoru Konnai

**Affiliations:** 1 Department of Disease Control, Faculty of Veterinary Medicine, Hokkaido University, Sapporo, Japan; 2 Business Development Unit, FASMAC Co., Ltd., Atsugi, Japan; 3 Molecular and Cellular Epigenetics, Interdisciplinary Cluster for Applied Genoproteomics (GIGA), University of Liège, Liège, Belgium; 4 Laboratory of Public Health, Faculty of Veterinary Medicine, Hokkaido University, Sapporo, Japan; 5 Veterinary Research Unit, International Institute for Zoonosis Control, Hokkaido University, Sapporo, Japan; 6 Institute for Vaccine Research and Development (HU-IVReD), Hokkaido University, Sapporo, Japan; 7 One Health Research Center, Hokkaido University, Sapporo, Japan; 8 Department of Antibody Drug Development, Tohoku University Graduate School of Medicine., Sendai, Japan; 9 Division of Bioresources, International Institute for Zoonosis Control, Hokkaido University, Sapporo, Japan; 10 Global Station for Zoonosis Control, Global Institution for Collaborative Research and Education (GI-CoRE), Hokkaido University, Sapporo, Japan; 11 International Affairs Office, Faculty of Veterinary Medicine, Hokkaido University, Sapporo, Japan

**Keywords:** B-cell lymphoma, BLV, CCL4, EBL, macrophage, TAM

## Abstract

In enzootic bovine leukosis (EBL), a B-cell lymphoma caused by bovine leukemia virus (BLV) infection, immune remodeling within tumor-affected lymph nodes is currently poorly understood. Here, we analyzed chemokine production and macrophage phenotypes in tumor-affected lymph nodes in EBL, focusing on CCL4. Intracellular cytokine staining revealed that, compared with healthy lymph nodes, EBL tumor-affected lymph nodes showed increased proportions of CCL4 expressing cells, particularly among CD4^+^ T cells and B cells. Functional migration assays showed that recombinant CCL4 induced robust monocyte migration, with a comparatively modest T-cell migration. To identify lymph node macrophages, we characterized CD11b^hi^CD172a^+^ cells and confirmed their macrophage identity based on high expression levels of CD11c, CD14, CD16, and CD68. Using this definition, we found that the CD11b^hi^CD172a^+^ population was markedly increased in tumor-affected lymph nodes. Phenotypic analysis revealed an increased proportion of CD163^+^ and CD163^+^PD-L1^+^ macrophages, consistent with the acquisition of an immunosuppressive phenotype. In parallel, macrophages in tumor-affected lymph nodes showed a shift from MHC class I/II^hi^ to MHC I/II^lo^ subsets and reduced co-expression of MHC molecules with CD80 and CD86, indicating impaired antigen-presenting features. Collectively, these findings suggest that elevated CCL4 production in tumor lesions induced by EBL is associated with monocyte recruitment and the accumulation of phenotypically altered macrophages, highlighting a chemokine-driven remodeling of the tumor immune microenvironment.

## 1 Introduction

Bovine leukemia virus (BLV) is a retrovirus that infects bovine B cells and induces the development of a B-cell lymphoma known as enzootic bovine leukosis (EBL) (1). Following BLV infection, infected B cells expand during the chronic phase, progressing from an alymphocytotic state to persistent lymphocytosis, and EBL can develop after long-term infection (2–4). This systemic lymphoma is characterized by a predominance of transformed BLV-infected B cells in lymph nodes throughout the body as well as in various non-lymphatic organs and is often accompanied by leukemic manifestations and ultimately death (5–7). Although viral factors and host immune responses have been implicated in EBL progression, the mechanisms underlying B-cell transformation and disease development remain poorly defined (4, 8, 9). Despite extensive efforts to elucidate the pathogenesis of BLV infection, and the development of multiple therapeutic and vaccine-based approaches in different countries worldwide (10, 11), no effective vaccine or curative therapy is currently commercially available. Therefore, a deeper understanding of the disease mechanisms underlying BLV-associated leukemogenesis and immune dysregulation is required to support the development of improved control strategies.

Accumulating evidence has demonstrated that T-cell exhaustion mediated by immune checkpoint molecules plays a critical role in BLV infection and disease progression (12). As the infection advances, the expression of multiple immune checkpoint molecules, including programmed death-1 (PD-1) (13, 14), PD-ligand 1 (PD-L1) (15), T-cell immunoglobulin domain and mucin domain-3 (TIM-3) (16, 17), cytotoxic T-lymphocyte-associated antigen-4 (CTLA-4) (18) and lymphocyte-activation gene-3 (LAG-3) (19, 20), increases, resulting in impaired T-cell effector functions. Notably, EBL-induced tumors are characterized by severely exhausted T cells co-expressing PD-1 and TIM-3, which exhibit markedly reduced responsiveness to T-cell stimulation (21). The accumulation of such severely exhausted T cells is one of the hallmarks of an immunosuppressive tumor microenvironment driven by persistent antigen exposure and inhibitory signaling (22, 23). However, the cellular and molecular mechanisms underlying the establishment of this immunosuppressive microenvironment in EBL remain largely unexplored.

Recent studies in cancer immunology have revealed that exhausted T cells do not simply lose effector functions but may undergo functional reprogramming (24, 25). In addition to diminished cytotoxicity and proliferative capacity, exhausted T cells can selectively retain or even enhance the production of specific inflammatory mediators and chemokines (26–29). Such selective functional preservation has been implicated in the active remodeling of the tumor microenvironment rather than in direct antitumor immunity. In human and mouse tumor models, enhanced chemokine production promotes the recruitment of myeloid cells, especially tumor-associated macrophages (TAMs), which frequently accumulate in tumors and acquire immunosuppressive phenotypes (30–32). TAMs are known to suppress antitumor immune responses through multiple mechanisms, including inhibition of T-cell activation (33, 34) and modulation of antigen presentation (35, 36), thereby contributing to tumor progression. In particular, the chemokine (C-C motif) ligand 4 (CCL4)–CCR5 axis has been reported to mediate the recruitment of monocytes and macrophages and to facilitate the formation of immunosuppressive tumor niches (31).

In BLV-infected cattle, increased *CCL4* gene expression has been reported in exhausted T cell fractions co-expressing PD-1 and TIM-3, suggesting a potential link between exhausted T cells, chemokine production, and remodeling of the tumor microenvironment (21). However, the cellular sources of CCL4, the composition of immune cells recruited by CCL4, and the contribution of macrophages to the EBL tumor microenvironment remain poorly defined.

In this study, we aimed to characterize CCL4-associated chemokine responses in tumor-affected lymph nodes and to examine their relationship with immune cell recruitment and macrophage phenotypes, in order to improve understanding of the immunological features of EBL.

## 2 Materials and methods

### 2.1 Bovine lymph node and tumor samples

Lymph nodes samples used in this study were collected from adult cattle at the Hokkaido Hayakita Meat Inspection Center (Abira, Hokkaido, Japan). Normal lymph nodes were collected from clinically healthy cattle without gross abnormalities, whereas tumor-affected lymph node samples were obtained from cattle showing grossly evident lymphomatous lesions. All samples were processed within 48 h after collection. All animal procedures were approved by the Ethics Committee of the Faculty of Veterinary Medicine, Hokkaido University (approval numbers 17–0024 and 22-0038). Lymph nodes were collected from cattle diagnosed with EBL and from healthy controls. In EBL cases, samples were obtained from multiple anatomical sites depending on tumor involvement, including internal iliac lymph node (iiLN), mediastinal lymph node (medLN), superficial cervical lymph node (ScLN), mesenteric lymph node (mLN), renal lymph node (renLN), and tumor lesions in the diaphragm. In healthy cattle, lymph nodes were collected from iiLN or mLN. Detailed information on individual animals and sampling sites is provided in **Supplementary Table 1**. Lymph node tissues were finely chopped with scissors and mechanically dissociated into single-cell suspensions using a 40-μm cell strainer (BD Biosciences, Franklin Lakes, NJ, USA). The cells were washed twice with phosphate-buffered saline (PBS) containing 0.5 mg/mL disodium EDTA (Dojindo Molecular Technologies, Kumamoto, Japan), cryopreserved in liquid nitrogen, and stored at −80 °C until analysis.

### 2.2 Diagnosis of BLV infection and EBL onset

BLV infection was confirmed by the detection of BLV provirus in DNA specimens using quantitative real-time PCR using a LightCycler 480 System II (Roche Diagnostics, Mannheim, Germany) with a BLV detection kit (TaKaRa Bio, Otsu, Japan) as described previously (37). Tumor-affected lymph nodes were further evaluated via histopathological examination. EBL cattle were defined as cattle manifesting lymphomas or leukemia accompanied by clonal expansion of BLV-infected cells. The clonal expansion was confirmed using the clonality analysis by RAISING as described previously (37).

### 2.3 Cell culture

Single cell suspensions from lymph nodes were suspended in RPMI1640 medium (Sigma–Aldrich, St. Louis, Missouri, USA) supplemented with 10% heat-inactivated FBS (Thermo Fisher Scientific, Waltham, Massachusetts, USA), 200 IU/mL of penicillin, 200 μg/mL of streptomycin, and 2mM L-glutamine (Thermo Fisher Scientific). The cells were then cultured in triplicate at 37 °C in 5% CO2 for 24 hours for intracellular staining of CCL4, 48 hours for the quantification of chemokines of supernatants. 1 ng/mL recombinant bovine IL-2 (Kingfisher Biotech, Saint Paul, Minnesota, USA) was added to support T cell viability.

### 2.4 ELISA

Culture supernatants of single cell suspensions of lymph nodes were collected, and the concentrations of CCL3, CCL4 and CCL5 were measured in duplicate using the BOVINE CCL3, CCL4 and CCL5 DO-IT-YOURSELF ELISA Kits (Kingfisher Biotech). Absorbance was measured at 450 nm using a microplate reader (MTP-900; Corona Electric, Tokyo, Japan). Cells were collected and analyzed intracellular expression of CCL4 by flow cytometry.

### 2.5 Blood collection

Peripheral blood from clinically healthy adult cattle (Holstein breed, female) was collected at the Field Science Center for the Northern Biosphere, Hokkaido University (Sapporo, Hokkaido, Japan). Peripheral blood mononuclear cells (PBMCs) were isolated from the blood samples using density-gradient centrifugation on 60% Percoll (GE Healthcare, Chicago, Illinois, USA). The experiments using bovine blood samples were approved by the Ethics Committee of the Faculty of Veterinary Medicine, Hokkaido University (approval numbers 17–0024 and 22-0038).

### 2.6 Migration assay

To analyze which immune cells are recruited by CCL4, PBMCs (5 × 10^5^) were added to top inserts containing 5.0 μm pore polycarbonate membrane (Corning Inc., Corning, New York, USA). Recombinant bovine CCL4 (Kingfisher Biotech) were added in bottom wells in RPMI-1640 medium. Migration through the membrane was analyzed after 3 hours of culture in 37 °C in 5% CO2. Cells were collected for analysis by flow cytometry. Absolute counting beads (Life Technologies) were added for quantification of the number of migrated cells. PBMCs were obtained from 11 individual cattle, and migration assays were performed in three independent experiments.

### 2.7 Flow cytometry

Flow cytometry was used to analyze the intracellular expression of CCL4, the expression of linage marker of macrophage in lymph nodes, phenotype of tumor associated macrophages. The antibodies used for staining are listed in **Supplementary Table 2**, and gating strategies are shown in **Supplementary Figures S1**, **4**, **9**, **11**. Fluorescence minus one (FMO) and unstained controls were used to define gating boundaries. The anti-bovine PD-L1 and TIM-3 monoclonal antibodies used in this study were generated as previously described (21, 38). To block nonspecific binding to Fc receptors, cells were preincubated with PBS containing 10% heat-inactivated goat serum (Thermo Fisher Scientific) at 25 °C for 15 minutes. Cells were then stained with surface antibodies at 25 °C for 20 minutes. After staining, cells were washed with PBS containing 0.5% bovine serum albumin (Sigma-Aldrich), 2 mM disodium EDTA (Dojindo Molecular Technologies, Kumamoto, Japan), and 0.1% sodium azide (Sigma-Aldrich). For intracellular chemokine staining, brefeldin A (10 µg/mL; Sigma-Aldrich)) was added 4 hours before staining. After surface staining, cells were fixed and permeabilized using BD Cytofix/Cytoperm solution (BD Biosciences, Franklin Lakes, New Jersey, USA) and then stained for intracellular markers CD79a and CD68, and intracellular CCL4. Samples were analyzed using a spectral cell analyzer SA3800 (SONY, Tokyo, Japan). Data analysis was conducted using FlowJo v10.4 software (BD Biosciences) or the SA3800 software (SONY).

### 2.8 Cell sorting

Fluorescence-activated cell sorting (FACS) was performed to sort CD4^+^T cells into exhausted (ex; PD-1^+^TIM-3^+^) and non-exhausted (non-ex; PD-1^−^TIM-3^−^, PD-1^+^TIM-3^−^, or PD-1^−^TIM-3^+^) CD4^+^T cells from dissociated lymph nodes from EBL cattle using a FACSAria II flow cytometer (BD Biosciences) as described in our previous study (21). The isolated cells were then cultured in triplicate at 37 °C in 5% CO_2_ either with 20 ng/μL phorbol 12-myristate 13-acetate (PMA; FUJIFILM Wako Pure Chemical, Osaka, Japan) and 1 μg/mL ionomycin (Sigma-Aldrich) for 18 h.

### 2.9 Reanalysis of RNA sequencing data

Previously generated bulk RNA sequencing (RNA-seq) data from exhausted (PD-1^+^TIM-3^+^) and non-exhausted CD4^+^ T-cell fractions were reanalyzed to examine transcriptional features associated with CCL4 expression. Differential expression analysis was performed using DESeq2 in R. In addition, gene set enrichment analysis (GSEA) was conducted using MSigDB Hallmark gene sets based on ranked gene lists. RNA-seq data used in this reanalysis were generated in our previous study (21) and are available in the DDBJ Sequence Read Archive under accession number PRJDB20193.

### 2.10 Immunofluorescent analysis

Lymph node cell suspensions derived from EBL samples were used. Adherent cells were obtained by plating cells in RPMI medium and incubating at 37 °C with 5% CO_2_ for 1 h, followed by removal of non-adherent cells by washing with PBS. Cells were fixed with 4% paraformaldehyde for 20 min at room temperature and permeabilized with 0.1% Triton X-100 in PBS for 20 min. Fc receptor blocking was performed using 10% goat serum for 15 min at room temperature. Immunofluorescence staining was performed using antibodies against CD172a (DH59B, Bio-Rad; 1:500), Iba-1 (Fujifilm Wako; 1:500), and CD79a (HM57, Bio-Rad; 1:200). Antibodies were labeled using Zenon anti-mouse IgG_1_ Alexa Fluor 488, Zenon anti-rabbit IgG Alexa Fluor 594, and Zenon anti-mouse IgG_1_ Alexa Fluor 647 (Thermo Fisher Scientific), respectively, and diluted in PBS prior to use. Samples were observed by confocal microscopy (Zeiss LSM 800; Carl Zeiss, Jena, Germany), and images were analyzed using Zen software (Carl Zeiss).

### 2.11 Statistics

Statistical analysis was conducted using R version 4.4.2. The Mann-Whitney U test was used and compare the concentration of chemokine in the culture supernatants, intracellular expression of CCL4, the population of the macrophage and expression levels of M1/M2 macrophage markers in lymph nodes between healthy and EBL cattle. The Wilcoxon signed-rank test was used to analyze the linage markers of macrophage. A paired t-test was conducted to evaluate the statistical significance of CCL4 production from PMA/ionomycin-stimulated T cells, which were cultured in triplicate. The Friedman test followed by the Wilcoxon signed-rank test was used to analyze the difference of migrated population of PBMCs between four groups of chemokine concentration. P values of less than 0.05 were considered significant.

## 3 Results

### 3.1 Enhanced CCL4 production by B cells and T cells in tumor-affected lymph nodes in EBL

**Figure 1:**
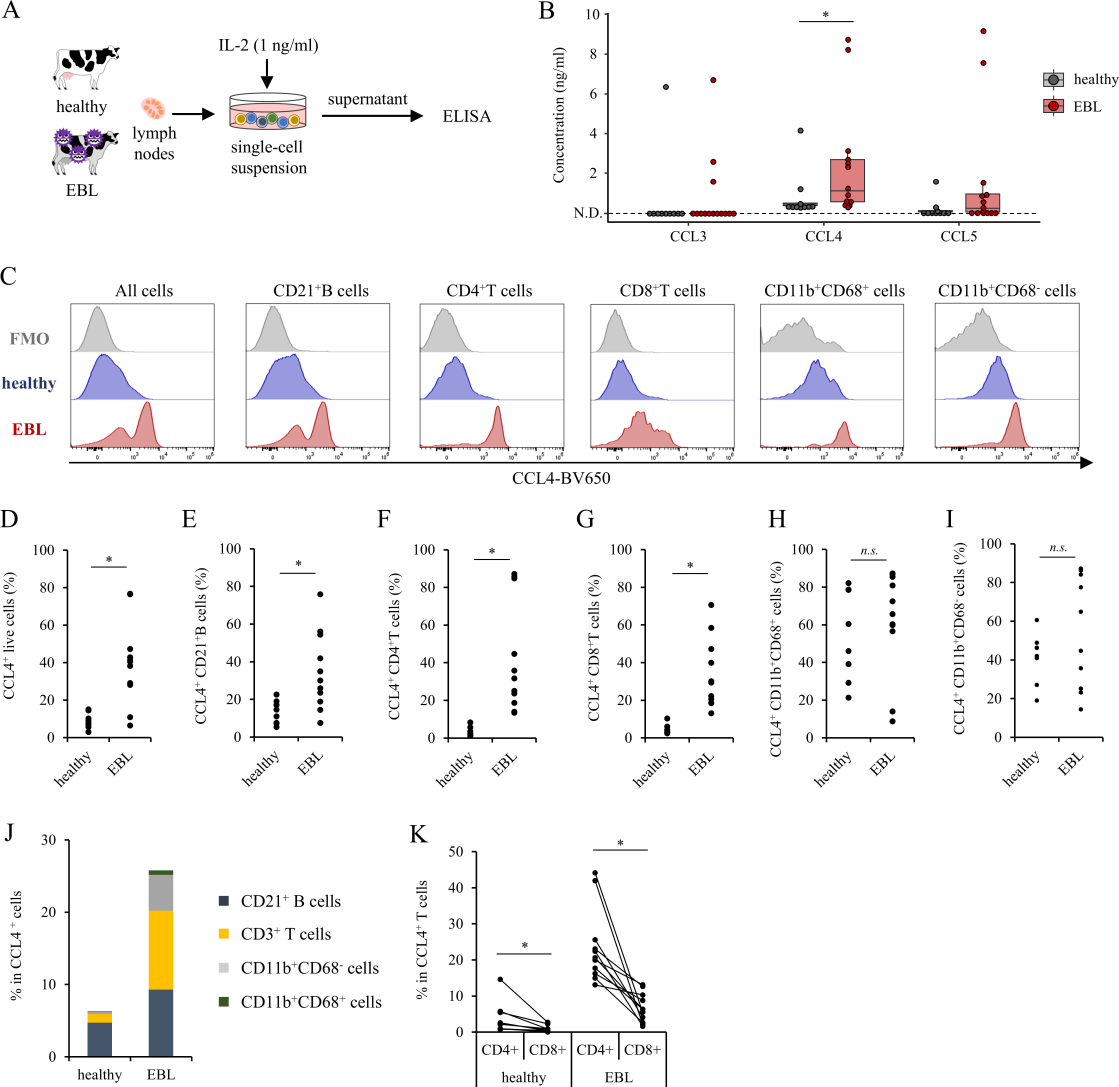
Comparison of cytokine and chemokine profiling in lymph nodes explants culture supernatant from healthy and EBL cattle **(A)** Schematic overview of the experimental design. Lymph nodes from healthy and EBL were cultured ex vivo, and culture supernatants were collected for cytokine and chemokine analysis, and cells were subjected to intracellular cytokine staining of CCL4. **(B)** Concentrations of CCL3, CCL4, and CCL5 in lymph node explant culture supernatants, measured by ELISA. Each dot represents an individual animal. N.D., not detected. **(C)** Histogram of CCL4 expression with fluorescent minus one (FMO) control. **(D–I)** Proportion of CCL4^+^ cells within individual immune cell subsets in healthy and EBL lymph nodes: **(D)** All live cells **(E)** B cells, **(F)** CD4^+^ T cells, **(G)** CD8^+^ T cells, **(H)** CD11b^+^CD68^-^ cells, **(I)** CD68^+^ cells. **(J)** Cellular composition of CCL4^+^ cells in healthy and EBL lymph nodes, summarized as median values and displayed as a stacked bar graph. **(K)** Proportions of CD4^+^ and CD8^+^ T cells within the CCL4^+^ T-cell population in EBL lymph nodes. *P < 0.05 (Mann–Whitney U test, Wilcoxon signed-rank test).

To compare chemokine production in lymph nodes, single-cell preparations from healthy and EBL lymph nodes were cultured and their supernatants were analyzed (**Figure 1A**). The analysis revealed that CCL4 production was significantly increased in EBL tumor-affected lymph nodes (hereafter referred to as “EBL lymph nodes”) compared with healthy lymph nodes (**Figure 1B**). In contrast, although CCL3 and CCL5 showed a tendency toward increased production in EBL lymph nodes, these changes did not reach statistical significance.

To identify the cellular sources of CCL4, intracellular staining was performed following 18 h of culture under the same conditions (**Supplementary Figures S1A, B**). The overall frequency of CCL4^+^ cells was significantly higher in EBL lymph nodes than in healthy lymph nodes (**Figures 1C, D**). Analysis of individual immune cell subsets revealed that CD21^+^ B cells (**Figures 1C, E**), CD4^+^ T cells (**Figures 1C, F**), and CD8^+^ T cells (**Figures 1C, G**) exhibited significantly higher expression of CCL4 in EBL lymph nodes. In contrast, CD11b^+^CD68^-^ cells and CD11b^+^CD68^+^ cells (myeloid cells including macrophages) did not show significant changes in CCL4 expression between healthy and EBL lymph nodes (**Figures 1C, H, I**). Analysis of the cellular composition of CCL4^+^ cells demonstrated that B cells and T cells constituted the dominant sources of CCL4 in EBL lymph nodes, whereas their contribution was markedly lower in healthy lymph nodes (**Figure 1J**; **Supplementary Figure S2**). Within the T-cell compartment, CCL4 expression in EBL lymph node was significantly higher in CD4^+^ T cells than in CD8^+^ T cells (**Figure 1K**).

### 3.2 Exhausted PD-1^+^TIM-3^+^ CD4^+^ T cells preferentially produce CCL4

**Figure 2:**
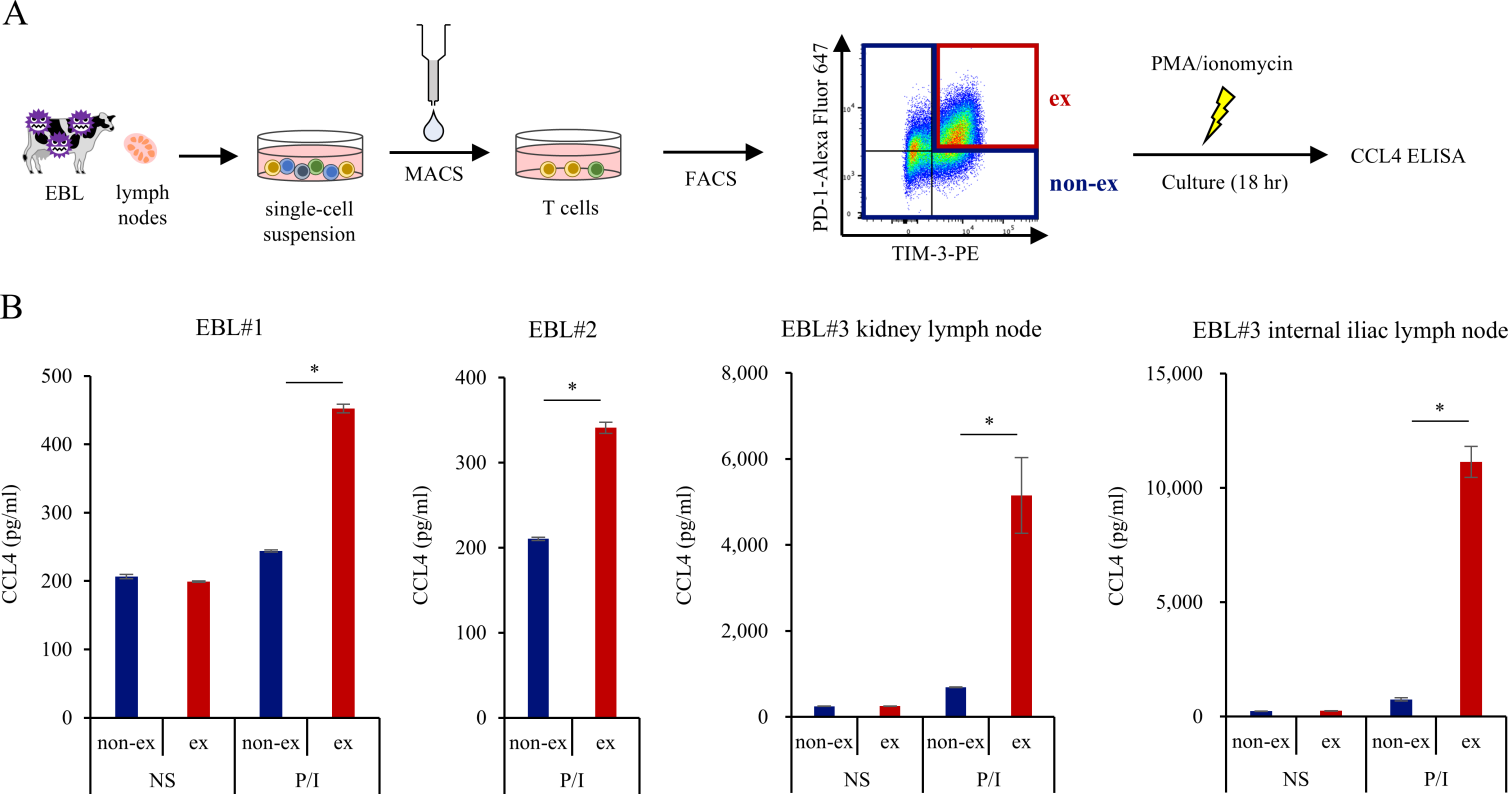
Upregulated CCL4 production in exhausted T cell fraction **(A)** Schematic representation of the sorting strategy used to isolate ex (exhausted; PD-1^+^TIM-3^+^) and non-ex (PD-1^−^TIM-3^−^, PD-1^+^TIM-3^−^, and PD-1^−^TIM-3^+^) CD4^+^ T-cell fractions from EBL tumor lymph nodes. **(B)** CCL4 production by sorted exhausted and non-exhausted CD4^+^ T-cell fractions from EBL tumor lymph nodes following stimulation with PMA (20 ng/µL) and ionomycin (1 μg/mL) for 18 h. Cells were cultured in triplicate, and CCL4 levels in culture supernatants were quantified by ELISA. EBL#1–3 represent samples obtained from three independent EBL cattle. The mean values of triplicates are shown in the bar. *n* = 9 (healthy) and *n* = 11 (EBL). *P < 0.05 (paired Student’s t test).

Based on our previous findings that exhausted PD-1^+^TIM-3^+^ CD4^+^T cells exhibit elevated *CCL4* expression at the RNA level (21), we next isolated exhausted and non-exhausted CD4^+^ T-cell fractions from EBL lymph nodes by cell sorting (**Figure 2A**). Sorted cells were stimulated with PMA (20 ng/µL) and ionomycin (1 μg/mL) for 18 h, and CCL4 concentrations in culture supernatants were quantified via ELISA. The result showed that exhausted CD4^+^ T cells produced significantly higher levels of CCL4 compared with non-exhausted CD4^+^ T-cell fractions (**Figure 2B**). To further support the enhanced CCL4 production observed in exhausted CD4^+^ T cells, previously generated RNA-seq data (21) were reanalyzed by comparing exhausted (PD-1^+^TIM-3^+^) and non-exhausted CD4^+^ T-cell fractions. Exhausted CD4^+^ T cells showed increased expression of CCL4 together with upregulation of exhaustion-related transcription factors TOX, TOX2, BATF, EOMES, whereas canonical TCR signaling components FOS/JUN, NFKB/RELA were decreased (**Supplementary Figure S3A**). Consistently, GSEA indicated negative enrichment of TNFα/NF-κB signaling (**Supplementary Figure S3B**).

Collectively, these results indicate that CCL4 production, predominantly driven by B cells and exhausted T cells, is markedly enhanced in EBL lymph nodes, suggesting a substantial remodeling of the immune microenvironment associated with EBL. Furthermore, the upregulation of CCL4 in exhausted T cells may occur independently of canonical TCR signaling.

### 3.3 CCL4 preferentially induces monocyte migration *in vitro*

**Figure 3:**
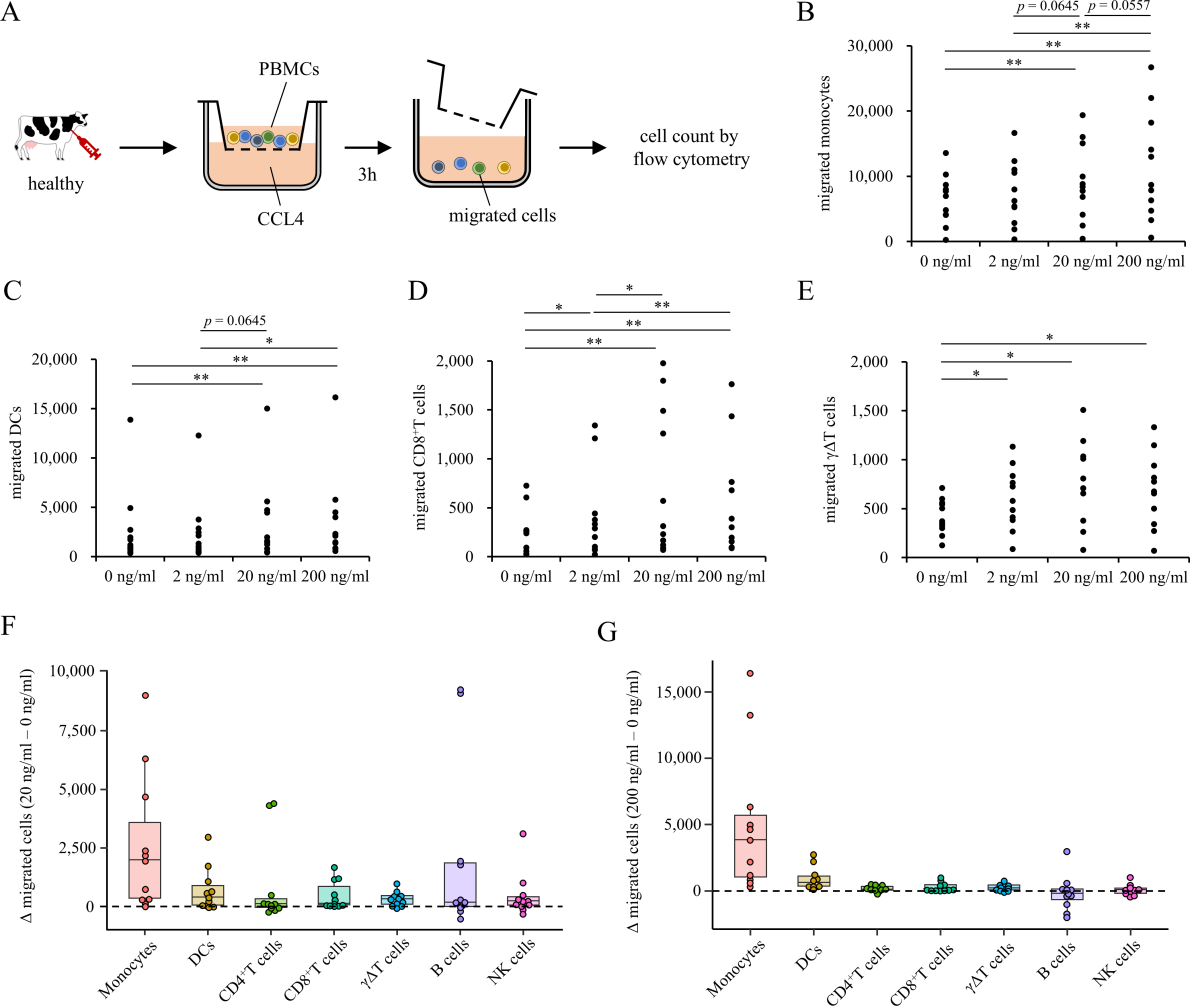
Transwell migration assay to evaluate immune cell responses to CCL4 **(A)** Schematic overview of the migration assay. PBMCs (5×10^5^) isolated from healthy cattle were placed in the upper insert, and recombinant bovine CCL4 was added to the lower chamber. After 3 h of incubation, migrated cells in the lower chamber were collected and quantified as absolute cell numbers by flow cytometry using counting beads. **(B–E)** Absolute numbers of migrated cells in response to increasing concentrations of CCL4 (0, 2, 20, and 200 ng/mL): **(B)** monocytes, **(C)** DCs, **(D)** CD8^+^ T cells, **(E)** γδ T cells. Each dot represents an independent experiment. **(F, G)** Background-subtracted migration of immune cell subsets at 20 ng/mL **(F)** and 200 ng/mL **(G)** CCL4. Values were calculated by subtracting the number of cells migrated in the absence of CCL4 (0 ng/mL) from those migrated in the presence of CCL4. Data are shown for monocytes, dendritic cells, CD4^+^ T cells, CD8^+^ T cells, γδ T cells, B cells, and NK cells. Data were obtained from PBMCs derived from 11 individual cattle across three independent experiments. *P < 0.05, **P < 0.01 (Friedman test followed by the Wilcoxon signed-rank test).

To investigate which immune cell populations are recruited by the increased CCL4 production observed in EBL lymph nodes, we performed transwell migration assays using recombinant bovine CCL4. Peripheral blood mononuclear cells (PBMCs) isolated from healthy cattle were placed in the upper chamber, while recombinant CCL4 was added to the lower chamber at concentrations of 2, 20, or 200 ng/mL. After 3 h of incubation, cells that migrated into the lower chamber were collected and quantified by flow cytometry (**Figure 3A**; **Supplementary Figure S4**). The results demonstrated that CCL4 induced a marked increase in cell migration at 20 and 200 ng/mL, particularly for CD14^+^CD11c^+^ monocytes (**Figure 3B**). In addition, at these concentrations, significant migration was observed for CD14^-^CD11c^+^ dendritic cells (DCs) (**Figure 3C**), CD8^+^ T cells (**Figure 3D**), and γδ T cells (**Figure 3E**). In contrast, migration of CD4^+^ T cells, B cells, and NK cells was minimal under the same conditions (**Supplementary Figures S5A–E**). To further comparatively analyze the relative contribution of each immune cell subset to CCL4-induced migration, background-subtracted migration values were calculated by subtracting the number of cells migrated in the absence of CCL4 (0 ng/mL) from those migrated in the presence of CCL4. At 20 and 200 ng/mL, monocytes exhibited the largest increase in migration compared with the other immune cell subsets (**Figures 3F, G**).

Overall, these results indicate that CCL4 preferentially promotes monocyte migration, while exerting additional effects on selected lymphocyte populations, suggesting that elevated CCL4 levels in EBL lymph nodes may contribute to the recruitment of monocytes into the tumor microenvironment.

### 3.4 Identification and expansion of macrophages in EBL lymph nodes

**Figure 4:**
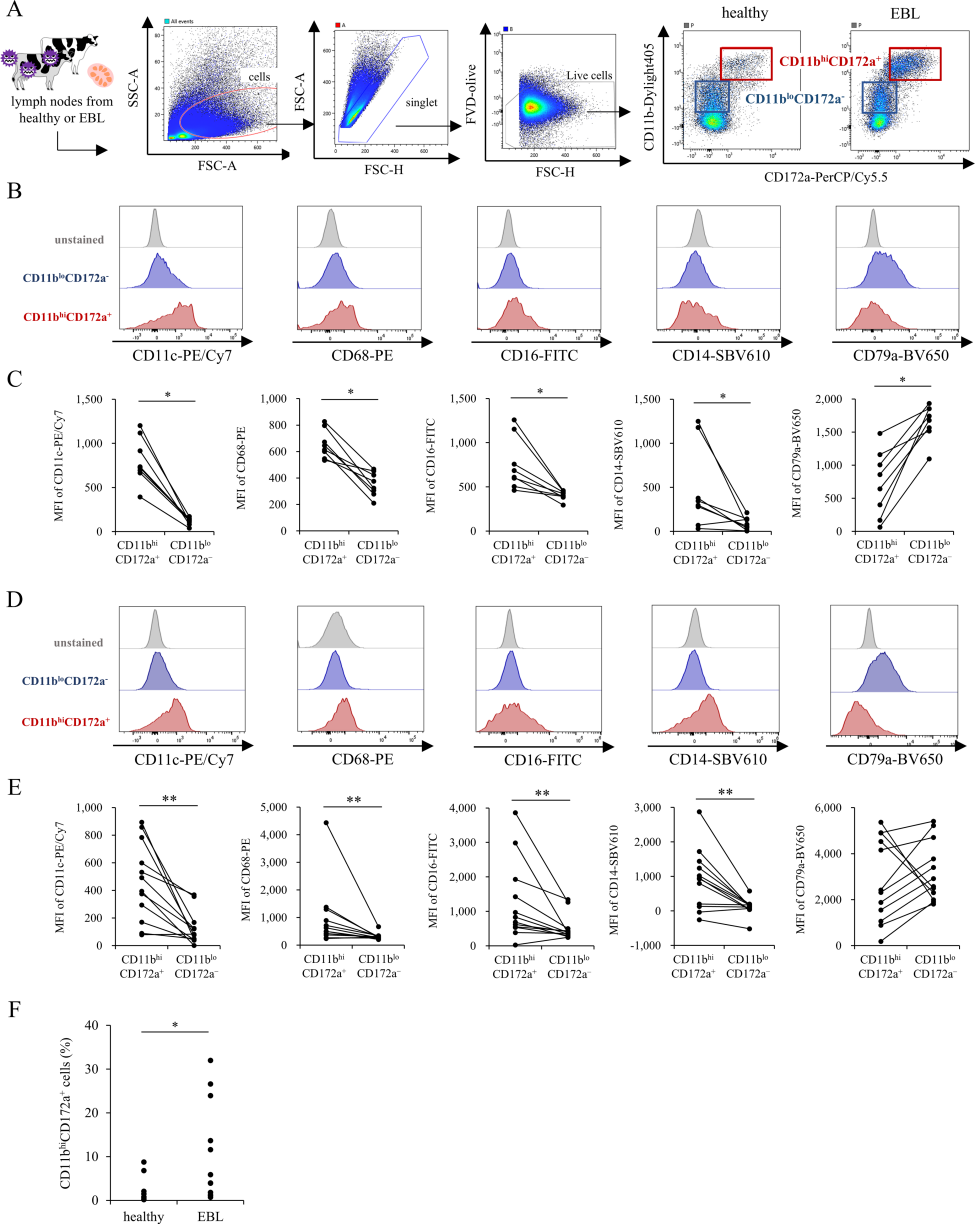
Identification of lymph node macrophages and comparison of their proportion in healthy and EBL lymph nodes. **(A)** Gating strategy for the identification of myeloid cell populations in bovine lymph nodes. Within CD11b^+^ cells, two distinct populations, CD11b^lo^CD172a^-^ and CD11b^hi^CD172a^+^, were identified. **(B)** Representative histograms showing the expression of CD11c, CD68, CD16, CD14 and CD79a in CD11b^hi^CD172a^+^ and CD11b^lo^CD172a^-^ cell populations in healthy lymph nodes. **(C)** Comparison of the MFI of CD11c, CD68, CD16, CD14, and CD79a in healthy lymph nodes. **(D)** Representative histograms showing the expression of CD11c, CD68, CD16, CD14 and CD79a in CD11b^lo^CD172a^-^ and CD11b^hi^CD172a^+^ cell populations in EBL lymph nodes. **(E)** Comparison of the MFI of CD11c, CD68, CD16, CD14, and CD79a in EBL lymph node. **(F)** Proportion of CD11b^hi^CD172a^+^ cells in healthy and EBL lymph nodes. *n* = 8 (healthy) and *n* = 12 (EBL). *P < 0.05 **P < 0.01 (Wilcoxon signed-rank test, Mann–Whitney U test).

We analyzed macrophage populations in lymph nodes from healthy and EBL cattle to determine whether monocytes/macrophages are increased in the EBL tumor microenvironment. Because macrophage phenotypes are highly tissue-specific and macrophage markers in bovine lymph nodes have not been well established, we first sought to identify lymph node macrophages in cattle. Single-cell suspensions from healthy bovine lymph nodes were analyzed by flow cytometry. Gating performed on myeloid markers CD11b and CD172a revealed two distinct CD11b^+^ populations: CD11b^lo^CD172a^-^ and CD11b^hi^CD172a^+^ cells (**Figure 4A**). To exclude neutrophils, cells with high side scatter were removed from the analysis. To characterize these two populations, we compared the expressions of macrophage-associated markers, such as CD11c, CD68, CD16, and CD14, as well as the B-cell marker CD79a. CD11b^hi^CD172a^+^ cells exhibited high expression of CD11c, CD68, CD16, and CD14, whereas CD11b^lo^CD172a^-^ cells showed higher expression of CD79a in healthy lymph nodes (**Figures 4B, C**). In line with this, the frequencies of CD11c^+^, CD68^+^, CD16^+^, and CD14^+^ cells were significantly higher within the CD11b^hi^CD172a^+^ population, whereas CD79a^+^ cells were enriched in the CD11b^lo^CD172a^-^ population (**Supplementary Figure S6A**).

This phenotypic pattern was similarly observed in EBL lymph nodes (**Figures 4D, E**; **Supplementary Figure S6B**). However, several individuals showed relatively high CD79a expression in CD11b^hi^CD172a^+^ cells. Notably, as CD79a was assessed by intracellular staining in flow cytometry, we hypothesized that its high expression in macrophages may reflect uptake of tumor B-cell antigens. To test this, we performed immunofluorescence staining in EBL lymph nodes to assess intracellular CD79a expression in macrophages. Consequently, CD79a expression was detected in a subset of CD172a^+^IBA-1^+^ macrophages (**Supplementary Figure S7**).

Based on these findings, CD11b^hi^CD172a^+^ cells were operationally defined as lymph node macrophages. Their proportion was significantly higher in EBL lymph nodes than in healthy controls. (**Figure 4F**). Collectively, these results demonstrate that CD11b^hi^CD172a^+^ macrophages are expanded in EBL lymph nodes and may represent TAM–like cells, consistent with the results of CCL4 migration assays.

### 3.5 Phenotypic alteration of tumor-associated macrophages in EBL lymph nodes

**Figure 5:**
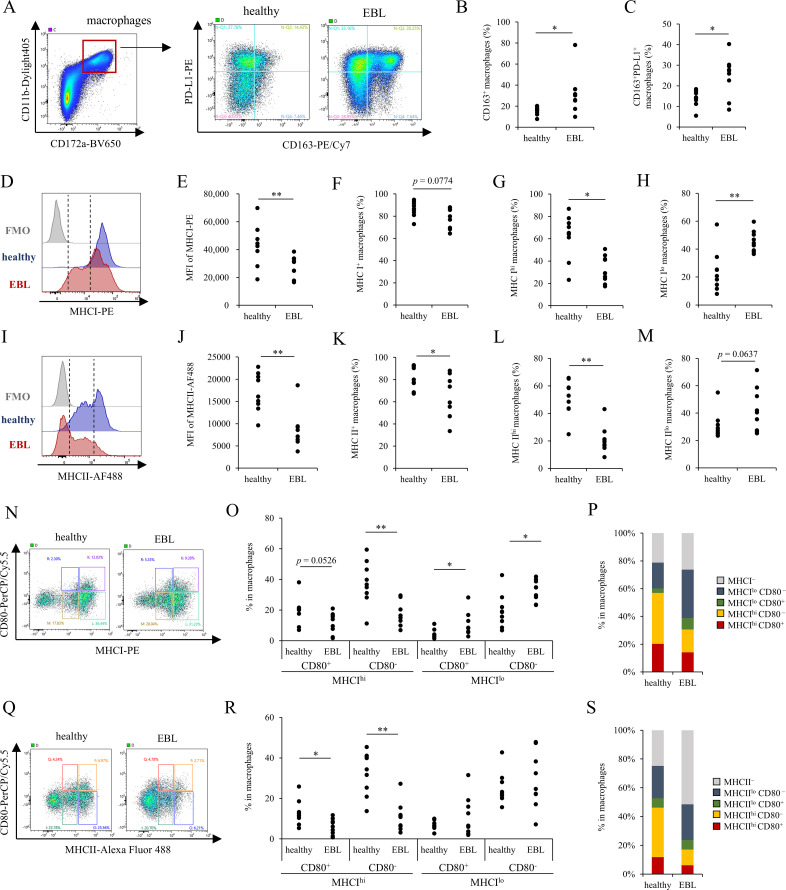
Phenotypic analysis of CD11b^hi^CD172a^+^ macrophages in healthy and EBL lymph nodes. **(A)** Representative flow cytometry plots showing CD163 and PD-L1 expression within macrophages (CD11b^hi^CD172a^+^) from lymph nodes. **(B)** Frequency of CD163^+^ cells among macrophages in healthy and EBL lymph nodes. **(C)** Frequency of CD163^+^PD-L1^+^ cells among macrophages in healthy and EBL tumor-bearing lymph nodes. **(D)** Representative histograms of MHC I expression in macrophages. **(E)** Proportions of MHC I^+^ macrophages. **(F, G)** Proportions of MHC I^hi^ **(F)** and MHC I^lo^ **(G)** subsets. **(H)** Representative plots showing the co-expression of MHC I and CD80 in macrophages. **(I)** Frequencies of CD80^+^ cells within MHC I^hi^ and MHC I^lo^ macrophage subsets in healthy and EBL lymph nodes. **(J)** Summary of the median distribution of CD80 co-expression within MHC I^hi^ and MHC I^lo^ macrophage subsets, displayed as a stacked bar graph. **(K)** Representative histograms of MHC II expression in macrophages. **(L)** Proportions of MHC II^+^ macrophages. **(M, N)** Proportions of MHC II^hi^ **(M)** and MHC II^lo^ **(N)** subsets. **(O)** Representative plots showing the co-expression of MHC II and CD80 in macrophages. **(P)** Frequencies of CD80^+^ cells within MHC II^hi^ and MHC II^lo^ macrophage subsets in healthy and EBL lymph nodes. **(Q)** Summary of the median distribution of CD80 co-expression within MHC II^hi^ and MHC II^lo^ macrophage subsets, displayed as a stacked bar graph. Data are shown for *n* = 9 animals per group. *P < 0.05, **P < 0.01 (Mann–Whitney U test).

To further characterize the phenotype of TAMs in EBL, we performed detailed phenotypic analyses of CD11b^hi^CD172a^+^ macrophages in lymph nodes from healthy cattle and EBL cattle. First, we examined the expression of markers associated with suppressive macrophage phenotypes. The proportions of CD163^+^ cells and CD163^+^PD-L1^+^ cells were significantly increased in EBL lymph nodes compared with healthy controls (**Figures 5A–C**). In contrast, no significant differences were observed in the expression of CD206, PD-L1, or TIM-3 alone between the two groups (**Supplementary Figures S8A–C**).

Next, to assess potential alterations in antigen-presentation related phenotypes, we analyzed the expression of major histocompatibility complex (MHC) class I and class II molecules present on TAMs, revealing reduced MHC class I expression levels in EBL lymph nodes, as indicated by decreased mean fluorescence intensity (MFI) (**Figures 5D–F**). Stratification of TAMs based on the intensity of MHC expression showed a decrease in the MHC I^hi^ population and a concomitant increase in the MHC I^lo^ population in EBL lymph nodes (**Figures 5G, H**). A similar shift was observed for MHC class II, with reduced frequencies of MHC II^hi^ macrophages and increased the frequencies of MHC II^lo^ macrophages in EBL lymph nodes (**Figures 5I–M**). Further analysis of the expression of costimulatory molecules CD80 and CD86, which are required for effective antigen presentation, did not reveal significant differences in overall expression levels between healthy and EBL lymph nodes (**Supplementary Figures S8D, E**). However, analysis of co-expression patterns showed that, in EBL lymph nodes, CD80 expression was reduced within the MHC I^hi^ macrophage population and relatively enhanced within the MHC I^lo^ population (**Figures 5N–P**; **Supplementary Figures S9A, B**). This redistribution of CD80 co-expression was even more pronounced when analyzed in relation to the expression of MHC class II molecules (**Figures 5Q–S**; **Supplementary Figures S9A, C**). Similar redistribution patterns were also observed for CD86, with reduced co-expression in MHC I/II^hi^ macrophages and relative enhancement in MHC I/II^lo^ subsets (**Supplementary Figures S9D, E**, **S10A–F**).

In parallel, we compared the expression of macrophage-associated markers between the CD11b^lo^CD172a^-^ and CD11b^hi^CD172a^+^ populations in healthy and EBL lymph nodes (**Supplementary Figure S11A**). The expression of macrophage-related molecules, including CD163, PD-L1, and TIM-3, was higher in CD11b^hi^ CD172a^+^ cells than in CD11b^lo^CD172a^-^ cells (**Supplementary Figures S11B, C**). In contrast, the levels of MHC class I/II expression were higher in the CD11b^lo^CD172a^-^ population than in CD11b^hi^ CD172a^+^ cells.

Collectively, these results demonstrate that TAMs in EBL lymph nodes exhibit altered expression of suppressive markers and a marked shift toward MHC^lo^ phenotypes, accompanied by changes in the distribution of costimulatory molecule expression.

## 4 Discussion

This study shows that tumor-affected lymph nodes in EBL cattle exhibited increased CCL4 production, with CCL4 derived mainly from T cells and B cells. Recombinant CCL4 preferentially induced monocyte migration, consistent with the accumulation of CD11b^hi^CD172a^+^ macrophages with immunosuppressive phenotypes in EBL lymph nodes. This indicates that CCL4-associated immune cell recruitment is linked to macrophage accumulation and an immunosuppressive tumor microenvironment in this type of B-cell lymphoma. Overall, these findings suggest that chemokine-driven immune cell recruitment contributes to the formation of an immunosuppressive environment in EBL, potentially linking exhausted T cells, tumor-transformed B cells, and macrophages.

Our intracellular staining analyses demonstrate that, within EBL lymph nodes, CCL4 is predominantly produced by CD4^+^ T cells and tumor-transformed B cells (**Figures 1C–K**). Generally, CCL4 is produced by activated T cells, monocytes, and other immune cells (39–41), and our results indicate that this is also the case in cattle. Exhausted CD4^+^ T cells retained a strong capacity for CCL4 production (**Figure 2**) despite their impaired effector function, consistent with the emerging view that exhausted T cells selectively maintain chemokine production (21, 27). Although CCL4 production by exhausted T cells has been reported in multiple solid tumors and is often linked to defined upstream signaling pathways, its molecular regulation appears to be context-dependent (42, 43). In the EBL setting, our RNA-seq reanalysis suggests that CCL4 upregulation in exhausted CD4^+^ T cells is accompanied by reduced expression of canonical TCR signaling components (**Supplementary Figure S3**), indicating that CCL4 production may occur independently of conventional activation pathways. These findings raise the possibility that, rather than being a simple byproduct of T-cell activation, CCL4 expression in exhausted T cells reflects a distinct transcriptional program associated with the exhausted state. In addition, Tumor-transformed B cells also exhibited increased CCL4 expression (**Figures 1E, J**), suggesting that neoplastic B cells themselves may actively contribute to shaping the tumor microenvironment. This raises the possibility that, in EBL, transformed B cells autonomously promote the recruitment of immunosuppressive myeloid cells through enhanced CCL4 production. In support of this notion, increased CCL4 expression by tumor-transformed B cells has been reported in chronic lymphocytic leukemia (CLL) (44, 45), diffuse large B-cell lymphoma (DLBCL) (46), and Epstein–Barr virus–associated B-cell malignancies (47, 48). In these settings, chronic BCR signaling or viral oncoproteins, such as EBNA2 or LMP1, activate SRC/SYK/BTK–NF-κB and MAPK/AP-1 pathways, promoting CCL4 transcription and secretion that supports tumor growth, survival, and microenvironmental remodeling. These observations raise the possibility that similar signaling mechanisms may contribute to enhanced CCL4 production by tumor-transformed B cells in EBL. Notably, substantial inter-individual variability in CCL4 production was observed across EBL samples, likely reflecting heterogeneity in the tumor microenvironment, including differences in the abundance and exhaustion status of T cells as well as the phenotypic diversity of tumor-transformed B cells.

The migration assay demonstrated that CCL4 preferentially induces the migration of CD14^+^CD11c^+^ monocytes, while also promoting the recruitment of CD14^-^CD11c^+^DC-like cells, CD8^+^ T cells, and CD4^+^ T cells (**Figure 3**). CCL3, CCL4, and CCL5 are known to induce chemotaxis mainly through CCR5 and in part through CCR1 (28), and the immune cell subsets that migrated in our assay were consistent with cell populations reported to express these receptors in other species. In the present study, direct assessment of CCR5 expression was not possible due to the lack of suitable bovine-specific antibodies. The availability of CCR5 as a marker in cattle would enable a more precise identification of CCL4-responsive immune cell subsets and substantially advance our understanding of CCL4-mediated formation of the tumor microenvironment in EBL. Among the migrated cell populations observed in this study, CD4^+^ and CD8^+^ T cells are known to exhibit functional impairment due to exhaustion in EBL (21), whereas, the phenotype and fate of recruited DCs could not be further examined in this study. Previous single-cell RNA-seq analyses of bovine lymph nodes have identified Flt3^+^ DC populations in healthy tissues, suggesting that DC subsets are present in cattle lymph nodes (49, 50). In this context, surface markers such as CD205 and CD1b, which have been suggested as useful identifiers of bovine lymph node DCs, may facilitate more detailed characterization of DC recruitment and function in EBL lymph nodes (51, 52). Together, these findings suggest that CCL4 broadly influences both lymphoid and myeloid cell recruitment within the tumor microenvironment. The preferential recruitment of monocytes further supports a role for CCL4 in shaping the myeloid compartment, including macrophages, in EBL. Although its causal role in macrophage recruitment *in vivo* remains to be determined, future studies incorporating functional perturbation approaches will be important to define the contribution of the CCL4 axis to immune regulation in EBL.

Accurate identification of macrophages in bovine lymph nodes via flow cytometry has been limited due to the scarcity of well-established lineage markers, such as CSF1R (53, 54) and CD64 (55, 56). These markers are commonly used to define TAM populations in humans and mice but are not readily applicable in cattle because of the lack of validated antibodies and inconsistent expression profiles. Previous bovine studies have proposed several candidate myeloid markers, including CD11b and CD172a, as useful identifiers of monocytes and macrophages (57). Accordingly, we defined lymph node macrophages as CD11b^hi^CD172a^+^ cells. Within this population, no further subset stratification was applied, and analyses were performed on the total CD11b^hi^CD172a^+^ compartment to ensure robust and unbiased comparison across samples, given the variability in marker expression. This cell population expressed canonical macrophage-associated markers such as CD11c, CD16, CD14, and CD68 (**Figure 4B**); however, in some individuals, a CD11b^hi^CD172a^+^ subset exhibited reduced expression of CD14 (**Figure 4C**, **Supplementary Figure S6A**). While this observation should be interpreted with caution because multiple comparisons were not adjusted in this analysis, it is nevertheless consistent with the possibility of tissue-specific adaptation, as macrophages residing within lymph nodes can downregulate certain myeloid markers following differentiation or tissue integration (50, 58). Notably, the CD11b^hi^CD172a^+^ population also showed low-level CD79a positivity, particularly in EBL lymph nodes (**Figures 4D, E**, **Supplementary Figure S6B**). Because CD79a staining was performed intracellularly, this signal may reflect phagocytosed BLV-infected B cells rather than true CD79a expression by macrophages. Consistent with this interpretation, confocal immunofluorescence analysis revealed intracellular CD79a signals within CD172a^+^Iba-1^+^ macrophages (**Supplementary Figure S7**). Macrophages are known to exhibit high autofluorescence and retain phagocytosed material intracellularly, which can complicate interpretation of intracellular staining (59, 60). In addition, downregulation of B-cell surface markers in EBL further limits the reliable exclusion of B cells based on surface phenotyping alone (61). Together, these factors highlight the need for improved marker strategies to accurately define macrophage populations in BLV-associated tumors.

Phenotypic analysis of CD11b^hi^CD172a^+^ cells demonstrated that TAMs in EBL lymph nodes exhibited an immunosuppressive phenotype, characterized by increased CD163^+^ and CD163^+^PD-L1^+^ populations (**Figures 5A–C**) and reduced coordinated expression of MHC class I/II molecules with costimulatory molecules such as CD80 and CD86 (**Figures 5N–S**, **Supplementary Figures S8D, E**, S9). This suggests that monocytes recruited into the tumor microenvironment undergo suppressive polarization accompanied by impaired antigen-presenting capacity, a phenotype known in human and murine tumors to promote T-cell exhaustion. Although the expansion of macrophages retaining low levels of MHC expression may appear to support antigen presentation, insufficient co-stimulatory signaling is likely to result in incomplete T-cell activation, which can instead promote T-cell dysfunction and exhaustion (62, 63). Because EBL tumor lymph nodes are largely composed of tumor-transformed B cells that retain MHC class I/II expression, CD11b^lo^CD172a^^-^^ cells with high CD79a expression, which are likely neoplastic B cells, may constitute a major antigen-presenting population within these lesions (**Supplementary Figures S11B, C**). Therefore, antigen presentation within EBL lymph nodes may be dominated by tumor-transformed B cells, whereas macrophages provide insufficient costimulatory support. Such chronic and incomplete antigen presentation is a well-established driver of T-cell exhaustion (64–66). Overall, these findings suggest that dysregulated antigen presentation, rather than simple immune suppression, plays a central role in shaping T-cell exhaustion in EBL. However, direct functional assessment of antigen presentation by macrophages remains technically challenging in cattle due to the limited availability of antigen-specific T-cell assays. Therefore, identifying the antigens presented in this context—potentially through proteomic approaches such as immunopeptidomics, ideally combined with functional T-cell assays (67, 68)—may therefore be critical for understanding EBL-associated immune dysfunction.

Previous studies of BLV infection have primarily focused on T-cell dysfunction, particularly the induction of T-cell exhaustion through immune checkpoint upregulation (12–21), with comparatively limited attention to the role of macrophages in disease progression. Notably, several reports describing reduced phagocytic activity (69, 70), while their role in the EBL tumor microenvironment remains largely unexplored. In the present study, we provide evidence that exhausted T cells, together with tumor-transformed B cells, may actively contribute to the tumor microenvironment through enhanced CCL4 production, thereby promoting the recruitment of monocytes/macrophages. This raises the possibility of a feed-forward loop in which exhausted T cells facilitate the accumulation of immunosuppressive myeloid cells, further reinforcing T-cell dysfunction. These findings suggest that macrophage recruitment may represent a previously underappreciated component of BLV-associated pathogenesis, particularly in the transition from persistent lymphocytosis to overt lymphoma formation in EBL. In this context, both exhausted T cells and tumor-transformed B cells may cooperatively shape a tumor-permissive microenvironment through chemokine-mediated mechanisms. Similar chemokine-driven recruitment of tumor-associated macrophages has been reported in B-cell lymphomas such as DLBCL, where chemokines including CCL2 and CCL5 contribute to macrophage accumulation (71, 72). In HTLV-1–associated adult T-cell leukemia/lymphoma, increased chemokine expression (73, 74) and accumulation of CD163^+^ TAMs (75) have also been described. Together, these observations provide a framework for integrating T-cell exhaustion, chemokine signaling, and myeloid cell dynamics in BLV-associated disease, and highlight a potential role for macrophages in driving EBL development.

By systematically identifying and phenotyping macrophages in EBL tumor-affected lymph nodes via flow cytometry, the present study addresses a major technical gap in bovine immunology and provides a basis for analyzing macrophage populations in EBL. Further investigation of heterogeneity of TAMs, their interactions with BLV-infected B cells and exhausted T cells, and chemokine receptor pathways such as the CCL4–CCR5 axis will be required to refine our understanding of immune regulation in EBL. Collectively, these findings expand our current knowledge of the immunological features of this B-cell lymphoma type and support future research aimed at dissecting tumor-associated immune mechanisms in cattle.

## Data Availability

The original contributions presented in the study are included in the article/**Supplementary Material**. Further inquiries can be directed to the corresponding author.

## Supplementary material

Supplementary Figure 1 Gating strategy for analysis of CCL4-producing cells in lymph nodes. **(A)** Gating strategy used to quantify the proportion of CCL4-producing cells within each immune cell population (lymphoid and myeloid lineages) in lymph node–derived single-cell suspensions. **(B)** Gating strategy used to determine the cellular composition of total CCL4^+^ cells, showing the relative contribution of each immune cell subset among CCL4-producing cells.

Supplementary Figure 2 Distribution of CCL4-producing cells and T-cell subsets across individual lymph nodes or tumor samples. **(A)** Proportion of CCL4-producing cells in each EBL tumor lymph node sample. **(B)** Proportions of CD4^+^ and CD8^+^ T cells within total T cells in each EBL tumor–derived lymph node sample. **(C)** Proportion of CCL4-producing cells in lymph nodes from healthy cattle. **(D)** Proportions of CD4^+^ and CD8^+^ T cells within total T cells in lymph nodes from healthy cattle. iiLN, internal iliac lymph node; medLN, mediastinal lymph node; diaphragm, tumor lesion in the diaphragm; ScLN, superficial cervical lymph node; mLN, mesenteric lymph node; renLN, renal lymph node.

Supplementary Figure 3 Transcriptomic features of exhausted CD4^+^ T cells. **(A)** Expression of CCL4-related genes in exhausted (PD-1^+^TIM-3^+^) and non-exhausted CD4^+^ T cells. **(B)** GSEA enrichment plot showing negative enrichment of TNFα/NF-κB signaling in exhausted CD4^+^ T cells.

Supplementary Figure 4 Gating strategy for quantification of migrated cells in the migration assay.

Supplementary Figure 5 Additional analyses of immune cell migration in response to CCL4. **(A)** Total number of migrated cells quantified in the migration assay. **(B)** Migration of total CD3+ T cells. **(C)** Migration of B cells. **(D)** Migration of NK cells. **(E)** Migration of CD4^+^ T cells. Data were obtained from PBMCs derived from 11 individual cattle across three independent experiments. **P* < 0.05, ***P* < 0.01 (Friedman test followed by the Wilcoxon signed-rank test).

Supplementary Figure 6 Identification of lymph node macrophages in EBL. Frequencies of CD11c, CD68, CD16, CD14 and CD79a in CD11bhiCD172a^+^ and CD11bloCD172a^-^ cell populations **(A)** in healthy lymph nodes, **(B)** in EBL lymph node. n = 8 (healthy) and n = 12 (EBL). **P* < 0.05 ***P* < 0.01 (Wilcoxon signed-rank test).

Supplementary Figure 7 Immunofluorescence analysis showing intracellular CD79a signals in macrophages. Representative confocal immunofluorescence images of CD79a^-^ and CD79a^+^ macrophages. Nuclei are shown in blue (Hoechst), CD172a in green, Iba-1 in magenta, and CD79a in cyan (scale bar: 5 μm).

Supplementary Figure 8 Phenotypic analysis of lymph node macrophages. Comparison of the frequencies of macrophages expressing individual markers in healthy and EBL lymph nodes. **(A)** CD206^+^ cells, **(B)** PD-L1^+^ cells, **(C)** TIM-3^+^ cells, **(D)** CD80^+^ cells, **(E)** CD86^+^ cells. Data represent the proportion of marker-positive cells within the CD11b^hi^CD172a^+^ macrophage population. Data are shown for *n* = 9 animals per group. **P* < 0.05 (Mann–Whitney U test).

Supplementary Figure 9 Gating strategy for analysis of MHC I/II and co-stimulatory molecules. **(A)** Gating strategy used to define CD11b^hi^CD172a^+^ macrophage populations. **(B–E)** Representative flow cytometry plots within CD11b^hi^CD172a^+^ macrophages from lymph nodes **(B)** showing MHC I and CD80 co-expression, showing **(C)** MHC I and CD86 co-expression, **(D)** MHC II and CD80 co-expression, **(E)** MHC II and CD86 co-expression.

Supplementary Figure 10 Altered co-expression of MHC I/II and CD86 in lymph node macrophages. **(A)** Distribution of macrophage subsets within the CD11b^hi^CD172a^+^ population in healthy and EBL tumor-bearing lymph nodes. **(B)** Representative plots showing the co-expression of MHC class I and CD86 in CD11b^hi^CD172a^+^ macrophages. **(C)** Frequencies of CD86^+^ cells within MHC I^hi^ and MHC I^lo^ macrophage subsets in healthy and EBL lymph nodes. **(D)** Summary of the median distribution of CD86 co-expression within MHC I^hi^ and MHC I^lo^ macrophage subsets, displayed as a stacked bar graph. **(E)** Representative plots showing the co-expression of MHC class II and CD86 in CD11b^hi^CD172a^+^ macrophages. **(F)** Frequencies of CD86^+^ cells within MHC II^hi^ and MHC II^lo^ macrophage subsets in healthy and EBL tumor-bearing lymph nodes. **(G)** Summary of the median distribution of CD86 co-expression within MHC II^hi^ and MHC II^lo^ macrophage subsets, displayed as a stacked bar graph. Data are shown for *n* = 9 animals per group. **P* < 0.05 ***P* < 0.01 (Mann–Whitney U test).

Supplementary Figure 11 Comparative expression intensity of macrophage-associated markers between CD11b^hi^CD172a^+^ and CD11b^lo^CD172a^-^ cell populations. **(A)** Gating strategy used to define CD11b^hi^CD172a^+^ and CD11b^lo^CD172a^-^ populations for macrophage phenotyping. **(B)** Comparison of marker expression intensities between CD11b^hi^CD172a^+^ and CD11b^lo^CD172a^-^ populations in healthy lymph nodes. **(C)** Comparison of marker expression intensities between CD11b^hi^CD172a^+^ and CD11b^lo^CD172a^-^ populations in EBL tumor lymph nodes.
