# Supplementary material for: Exhausted CD4+T cells are associated with CCL4-driven immunosuppressive macrophage accumulation in enzootic bovine leukosis
Source: Front Immunol. 2026 May 4;17:1808815. doi: 10.3389/fimmu.2026.1808815 (PMC13180550; doi:10.3389/fimmu.2026.1808815)
Supplement: Supplementary Figure 1 — Gating strategy for analysis of CCL4-producing cells in lymph nodes. (A) Gating strategy used to quantify the proportion of CCL4-producing cells within each immune cell population (lymphoid and myeloid lineages) in lymph node–derived single-cell suspensions. (B) Gating strategy used to determine the cellular composition of total CCL4+ cells, showing the relative contribution of each immune cell subset among CCL4-producing cells. [file DataSheet1.pdf]

Supplementary Table 1. Summary of animals and lymph node sampling sites

| Case ID | Age (month) | Sex | Breed | Site |
|---|---|---|---|---|
| EBL#1 | 72 | Female | Holstein | ScLN |
| EBL#2 | 76 | Female | Holstein | medLN |
| EBL#3 | 132 | Female | Japanese black | iiLN, medLN, kidneyLN |
| EBL#4 | 99 | Female | Japanese black | medLN, ScLN, iiLN |
| EBL#5 | 82 | Female | Japanese black | medLN |
| EBL#6 | 69 | Female | Japanese black | iiLN, diaphragm |
| EBL#7 | 108 | Female | Japanese black | medLN, kidneyLN |
| EBL#8 | 30 | Male | Japanese black | iiLN |
| EBL#9 | 38 | Female | Japanese black | iiLN |
| EBL#10 | 120 | Female | Japanese black | iiLN |
| EBL#11 | 106 | Female | Japanese black | iiLN |
| EBL#12 | 77 | Female | Japanese black | iiLN |
| EBL#13 | 72 | Female | Holstein | mLN |
| EBL#14 | 56 | Female | Holstein | iiLN |
| EBL#15 | 53 | Female | Holstein | iiLN |
| EBL#16 | 27 | Male | Japanese black | kidneyLN |
| EBL#17 | 62 | Female | Japanese black | lungLN |
| EBL#18 | 28 | Male | Japanese black | iiLN |
| EBL#19 | 77 | Female | Japanese black | iiLN |
| EBL#20 | 31 | Female | Holstein | iiLN |
| healthy#1 | 90 | Female | Holstein | mLN |
| healthy#2 | 47 | Female | Holstein | mLN |
| healthy#3 | 48 | Female | Holstein | iiLN |
| healthy#4 | 63 | Female | Holstein | mLN, iiLN |
| healthy#5 | 77 | Female | Holstein | iiLN |
| healthy#6 | 71 | Female | Holstein | mLN |
| healthy#7 | 44 | Female | Holstein | mLN, iiLN |
| healthy#8 | 78 | Female | Holstein | iiLN |

# Supplementary Table 2. Antibody list for flow cytometry

| Target | Isotype | Clone | Fluorochrome | Source | Conjugation or labeling |
|---|---|---|---|---|---|
| **Expression analysis of intracellular CCL4** | | | | | |
| Dead cell | - | - | Olive | Thermo Fisher Scientific | LIVE/DEAD Fixable Olive (557) Viability Kit |
| CD3 | Mouse $IgG_1$ | MM1A | PerCp/Cy5.5 | WSU Monoclonal Antibody Center | Lightning-Link PerCp/Cy5.5 Conjugation Kit (Abcam) |
| CD4 | Mouse $IgG_{2a}$ | CC8 | PE/Cy7 | Bio-Rad | Lightning-Link PE/Cy7 Conjugation Kit (Abcam) |
| CD8 | Mouse $IgG_{2a}$ | CC63 | Pacific Blue | Bio-Rad | (Conjugated antibody product) |
| CD11b | Mouse $IgG_{2b}$ | CC126 | Dylight 405 | Bio-Rad | Lightning-Link Dylight 405 Conjugation Kit (Abcam) |
| CD68 | Mouse $IgG_1$ | ED1 | PE | Bio-Rad | (Conjugated antibody product) |
| CD79a | Mouse $IgG_1$ | HM57 | Alexa Fluor 488 | Bio-Rad | Zenon Alexa Fluor 488 Mouse $IgG_1$ Labeling Kit (Thermo Fisher Scientific) |
| CCL4 | Goat Ig | Polyclonal | Brilliant Violet 650 | Kingfisher Biotech | (Biotinylated antibody product) & Brilliant Violet 650 Streptavidin (BioLegend) |
| **Migration assay** | | | | | |
| Dead cell | - | - | Olive | Thermo Fisher Scientific | LIVE/DEAD Fixable Olive (557) Viability Kit |
| CD3 | Rat $IgG_1$ | CD3-12 | Alexa Fluor 488 | Bio-Rad | (Conjugated antibody product) |
| CD4 | Mouse $IgG_{2a}$ | CC8 | PE/Cy7 | Bio-Rad | Lightning-Link PE/Cy7 Conjugation Kit (Abcam) |
| CD8 | Mouse $IgG_{2a}$ | CC63 | Pacific Blue | Bio-Rad | (Conjugated antibody product) |
| CD11c | Mouse $IgG_1$ | BAQ153A | Dylight 405 | WSU Monoclonal Antibody Center | Lightning-Link Dylight 405 Conjugation Kit (Abcam) |
| CD14 | Mouse $IgG_1$ | CC-G33 | StarBright 610 | Bio-Rad | (Conjugated antibody product) |
| CD21 | Mouse $IgG_1$ | GB25A | Alexa Fluor 488 | WSU Monoclonal Antibody Center | Zenon Alexa Fluor 488 Mouse $IgG_1$ Labeling Kit (Thermo Fisher Scientific) |
| CD335 | Mouse $IgG_1$ | AKS1 | PE | Bio-Rad | Zenon PE Mouse $IgG_1$ Labeling Kit (Thermo Fisher Scientific) |
| **Identification of linage marker of macrophages in LNs** | | | | | |
| Dead cell | - | - | Olive | Thermo Fisher Scientific | LIVE/DEAD Fixable Olive (557) Viability Kit |
| CD11b | Mouse $IgG_{2b}$ | CC126 | Dylight 405 | Bio-Rad | Lightning-Link Dylight 405 Conjugation Kit (Abcam) |
| CD11c | Mouse $IgG_1$ | BAQ153A | PE/Cy7 | WSU Monoclonal Antibody Center | Lightning-Link PE/Cy7 Conjugation Kit (Abcam) |
| CD14 | Mouse $IgG_1$ | CC-G33 | StarBright 610 | Bio-Rad | (Conjugated antibody product) |
| CD16 | Mouse $IgG_{2a}$ | KD1 | FITC | AbD Serotec | (Conjugated antibody product) |
| CD68 | Mouse $IgG_1$ | ED1 | PE | Bio-Rad | (Conjugated antibody product) |
| CD172a | Mouse $IgG_1$ | DH59B | PerCp/Cy5.5 | Bio-Rad | PerCP/Cy5.5-conjugated anti-mouse IgG donkey antibody (BioLegend) |
| CD79a | Mouse $IgG_1$ | HM57 | Brilliant Violet 650 | Bio-Rad | Zenon Biotin Mouse $IgG_1$ Labeling Kit (Thermo Fisher Scientific) & Brilliant Violet 650 Streptavidin (BioLegend) |
| **M1 phenotyping of tumor associated macrophages** | | | | | |
| Dead cell | - | - | Olive | Thermo Fisher Scientific | LIVE/DEAD Fixable Olive (557) Viability Kit |
| CD11b | Mouse $IgG_{2b}$ | CC126 | Dylight 405 | Bio-Rad | Lightning-Link Dylight 405 Conjugation Kit (Abcam) |
| CD172a | Mouse $IgG_1$ | DH59B | Brilliant Violet 650 | Bio-Rad | Zenon Biotin Mouse $IgG_1$ Labeling Kit (Thermo Fisher Scientific) & Brilliant Violet 650 Streptavidin (BioLegend) |
| CD14 | Mouse $IgG_1$ | CC-G33 | StarBright Violet 610 | Bio-Rad | (Conjugated antibody product) |
| CD80 | Mouse $IgG_1$ | IL-A159 | PerCp/Cy5.5 | Bio-Rad | Lightning-Link PerCp/Cy5.5 Conjugation Kit (Abcam) |
| CD86 | Mouse $IgG_1$ | IL-A190 | PE/Cy7 | Bio-Rad | Lightning-Link PE/Cy7 Conjugation Kit (Abcam) |
| MHC class I | Mouse $IgG_{2a}$ | IL-A88 | PE | Thermo Fisher Scientific | Zenon PE Mouse $IgG_{2a}$ Labeling Kit (Thermo Fisher Scientific) |
| MHC class II | Mouse $IgG_{2a}$ | IL-A21 | Alexa Fluor 488 | Bio-Rad | Zenon Alexa Fluor 488 Mouse $IgG_{2a}$ Labeling Kit (Thermo Fisher Scientific) |
| **M2 phenotyping of tumor associated macrophages** | | | | | |
| Dead cell | - | - | Olive | Thermo Fisher Scientific | LIVE/DEAD Fixable Olive (557) Viability Kit |
| CD11b | Mouse $IgG_{2b}$ | CC126 | Dylight 405 | Bio-Rad | Lightning-Link Dylight 405 Conjugation Kit (Abcam) |
| CD172a | Mouse $IgG_1$ | DH59B | Brilliant Violet 650 | Bio-Rad | Zenon Biotin Mouse $IgG_1$ Labeling Kit (Thermo Fisher Scientific) & Brilliant Violet 650 Streptavidin (BioLegend) |
| CD163 | Mouse $IgG_1$ | BAQ128A | PE/Cy7 | WSU Monoclonal Antibody Center | Lightning-Link PE/Cy7 Conjugation Kit (Abcam) |
| CD206 | Mouse $IgG_1$ | 15-2 | Pacific Blue | BioLegend | (Conjugated antibody product) |
| PD-L1 | Rat $IgG_{2a}$ | 6C11 | PE | In house (38) | PE-conjugated anti-rat Ig goat antibody (Southern Biotech) |
| TIM-3 | Mouse $IgG_1$ | 4C2 | Alexa Fluor 488 | In house (21) | Zenon Alexa Fluor 488 Mouse $IgG_1$ Labeling Kit (Thermo Fisher Scientific) |

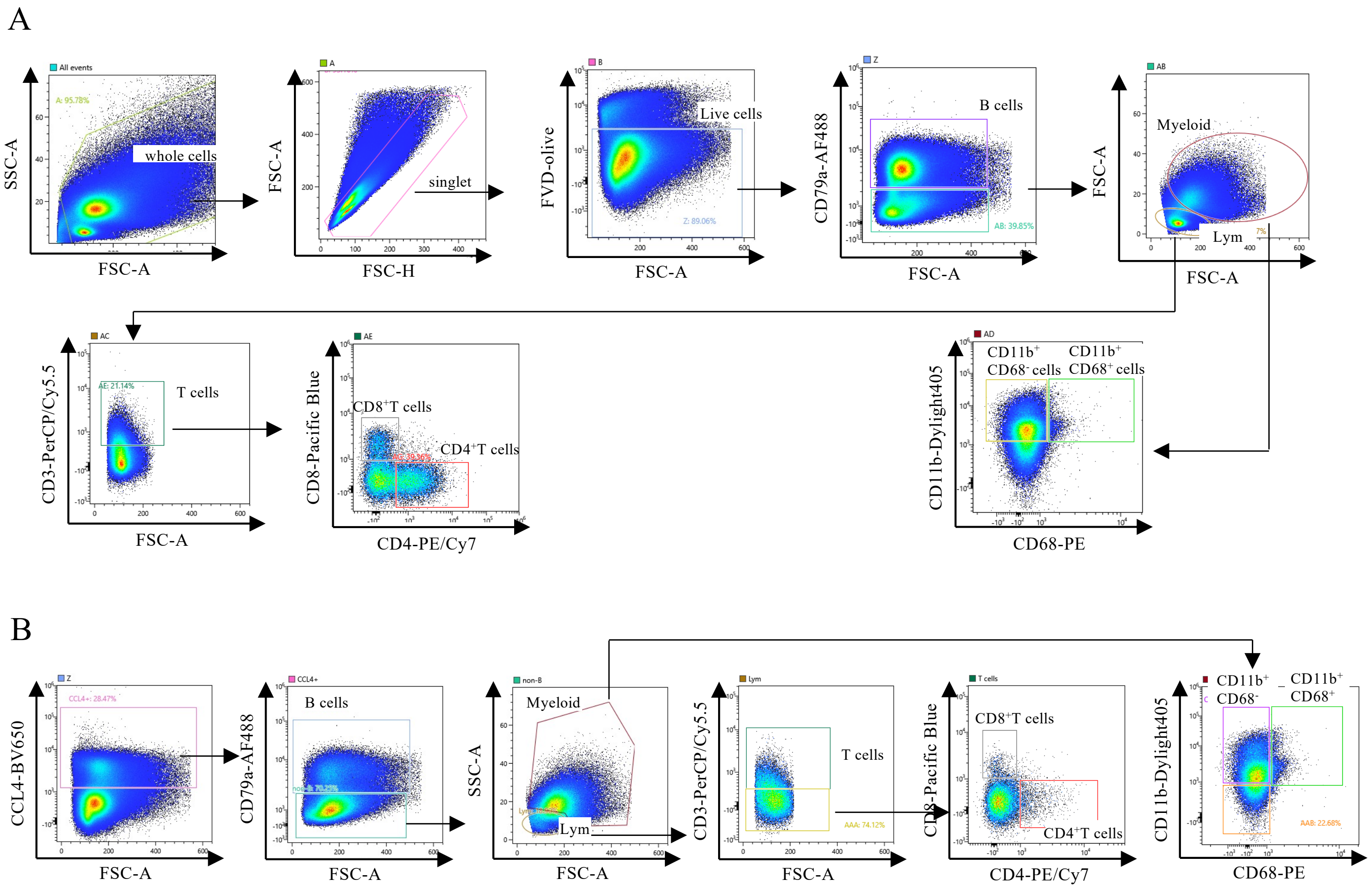


**Supplementary Figure 1. Gating strategy for analysis of CCL4-producing cells in lymph nodes**
(A) Gating strategy used to quantify the proportion of CCL4-producing cells within each immune cell population (lymphoid and myeloid lineages) in lymph node–derived single-cell suspensions. (B) Gating strategy used to determine the cellular composition of total CCL4$^{+}$ cells, showing the relative contribution of each immune cell subset among CCL4-producing cells.

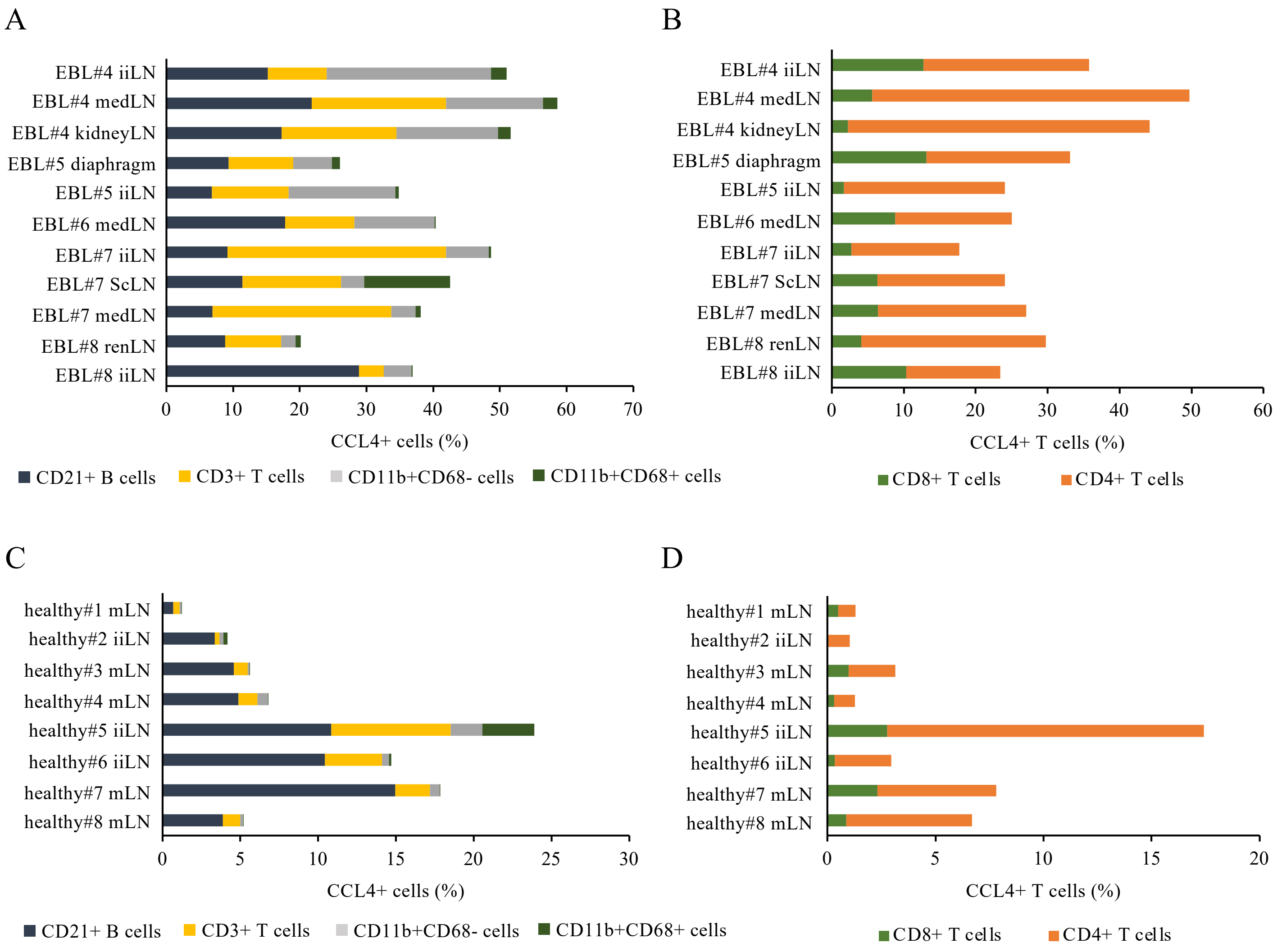


**Supplementary Figure 2. Distribution of CCL4-producing cells and T-cell subsets across individual lymph nodes or tumor samples**

(A) Proportion of CCL4-producing cells in each EBL tumor lymph node sample. (B) Proportions of CD4$^{+}$ and CD8$^{+}$ T cells within total T cells in each EBL tumor–derived lymph node sample. (C) Proportion of CCL4-producing cells in lymph nodes from healthy cattle. (D) Proportions of CD4$^{+}$ and CD8$^{+}$ T cells within total T cells in lymph nodes from healthy cattle. iiLN, internal iliac lymph node; medLN, mediastinal lymph node; diaphragm, tumor lesion in the diaphragm; ScLN, superficial cervical lymph node; mLN, mesenteric lymph node; renLN, renal lymph node.

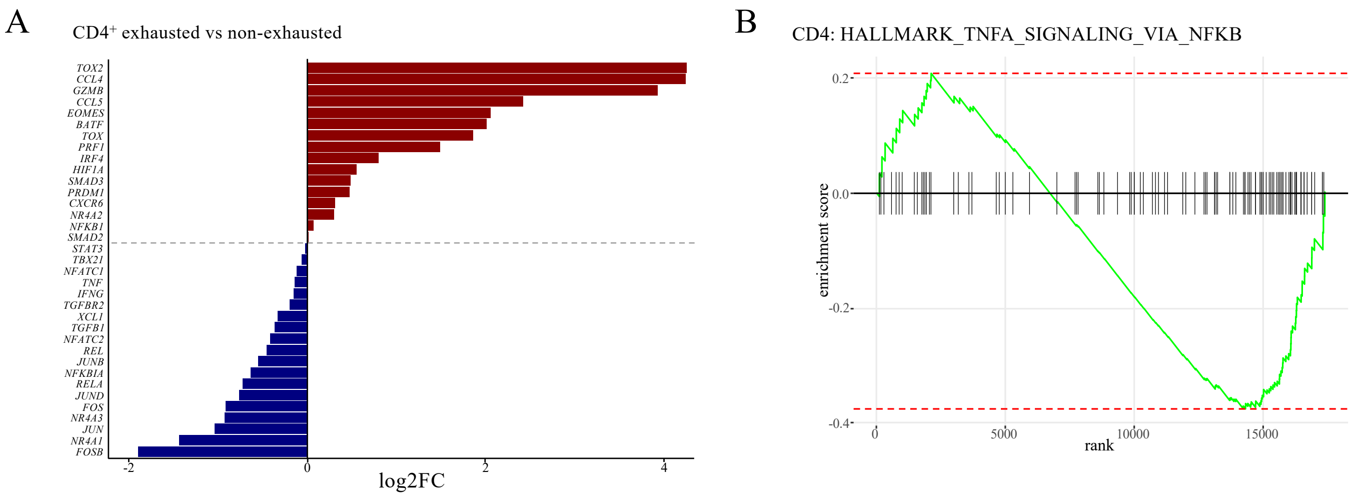


**Supplementary Figure 3. Transcriptomic features of exhausted CD4$^+$ T cells.**
(A) Expression of CCL4-related genes in exhausted (PD-1$^+$TIM-3$^+$) and non-exhausted CD4$^+$ T cells. (B) GSEA enrichment plot showing negative enrichment of TNFα/NF-κB signaling in exhausted CD4$^+$ T cells.

Supplementary Fig. 3

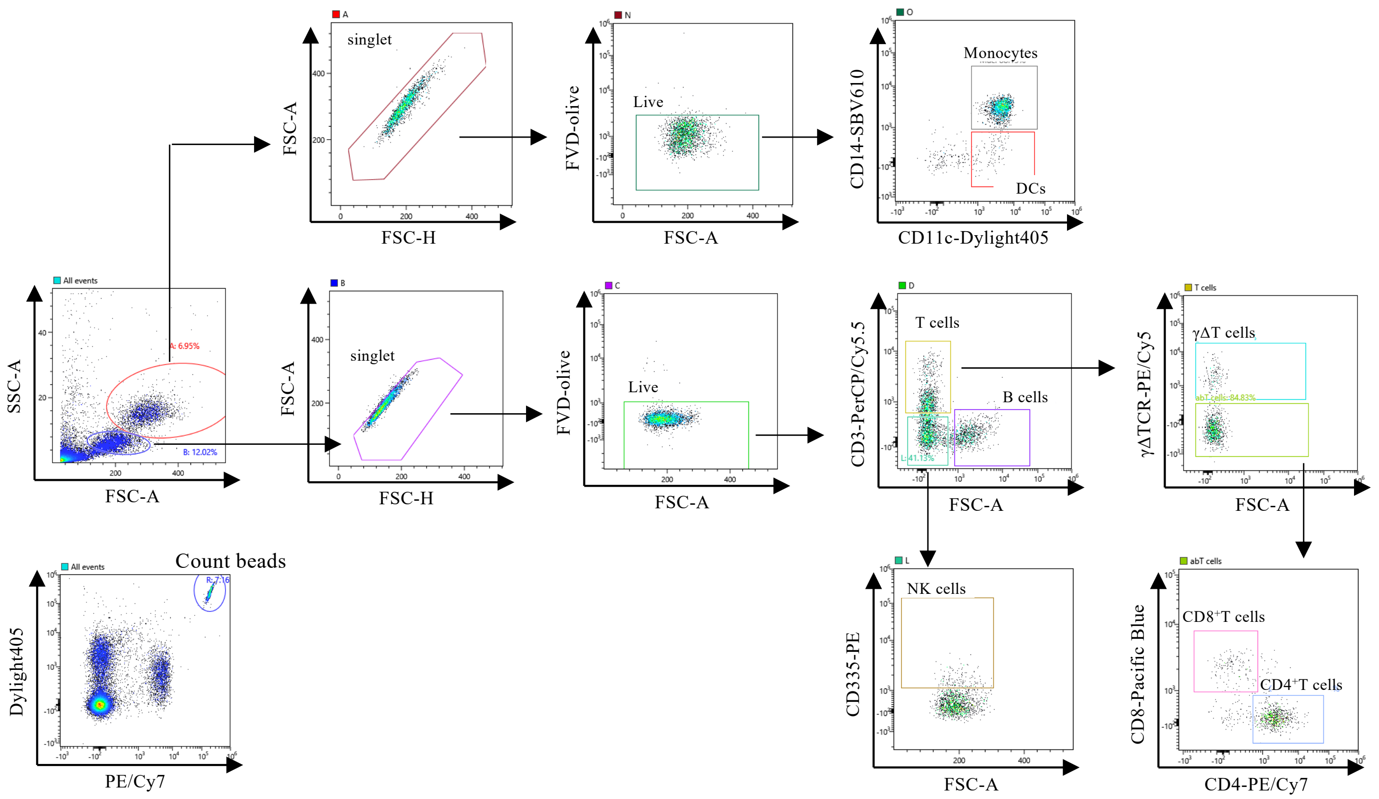


**Supplementary Figure 4. Gating strategy for quantification of migrated cells in the migration assay.**

Supplementary Fig. 4

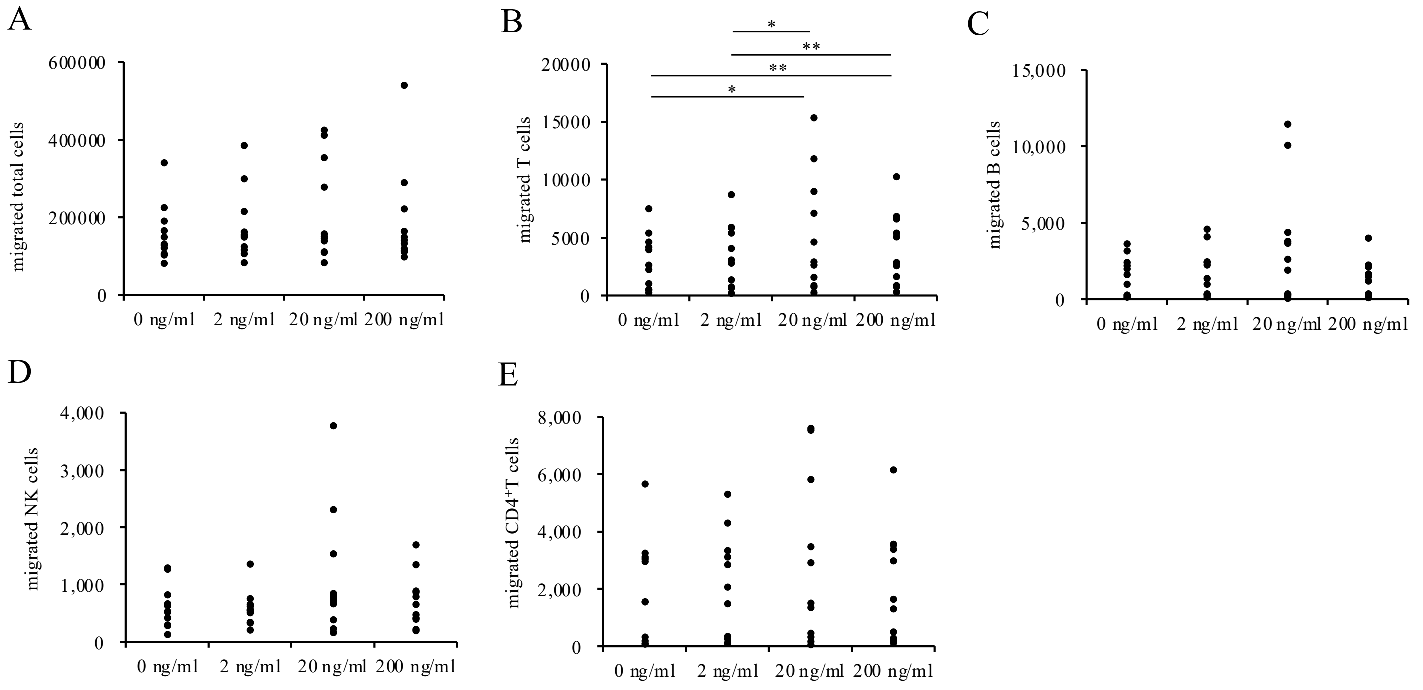


**Supplementary Figure 5. Additional analyses of immune cell migration in response to CCL4**

(A) Total number of migrated cells quantified in the migration assay. (B) Migration of total CD3+ T cells. (C) Migration of B cells. (D) Migration of NK cells. (E) Migration of $CD4^+$ T cells. Data were obtained from PBMCs derived from 11 individual cattle across three independent experiments. *$P < 0.05$, **$P < 0.01$ (Friedman test followed by the Wilcoxon signed-rank test).

Supplementary Fig. 5

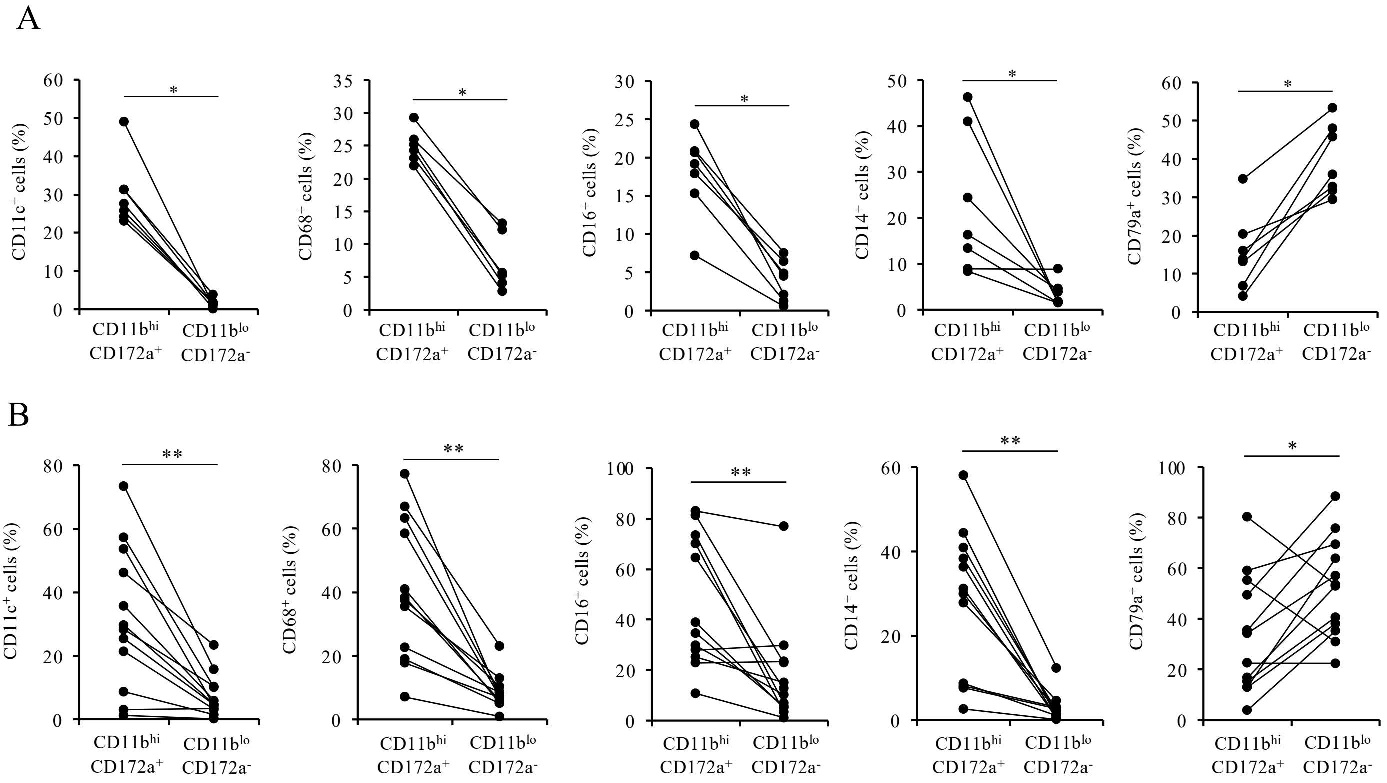


**Supplementary Figure 6. Identification of lymph node macrophages in EBL**

Proportion of CD11c, CD68, CD16, CD14 and CD79a expressing cells in $CD11b^{hi}CD172a^{+}$ and $CD11b^{lo}CD172a^{-}$ cell populations in (A) healthy lymph nodes, (B) EBL lymph node. $n$ = 8 (healthy) and $n$ = 12 (EBL). *$P$ < 0.05 **$P$ < 0.01 (Wilcoxon signed-rank test).

Supplementary Fig. 6

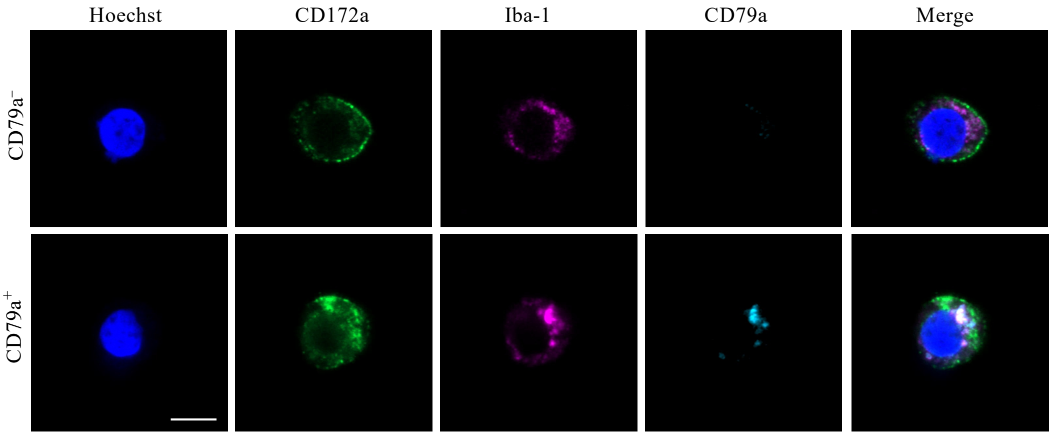


**Supplementary Figure 7. Immunofluorescence analysis showing intracellular CD79a signals in macrophages**

Representative confocal immunofluorescence images of $CD79a^-$ and $CD79a^+$ macrophages. Nuclei are shown in blue (Hoechst), CD172a in green, Iba-1 in magenta, and CD79a in cyan (scale bar: 5 µm).

Supplementary Fig. 7

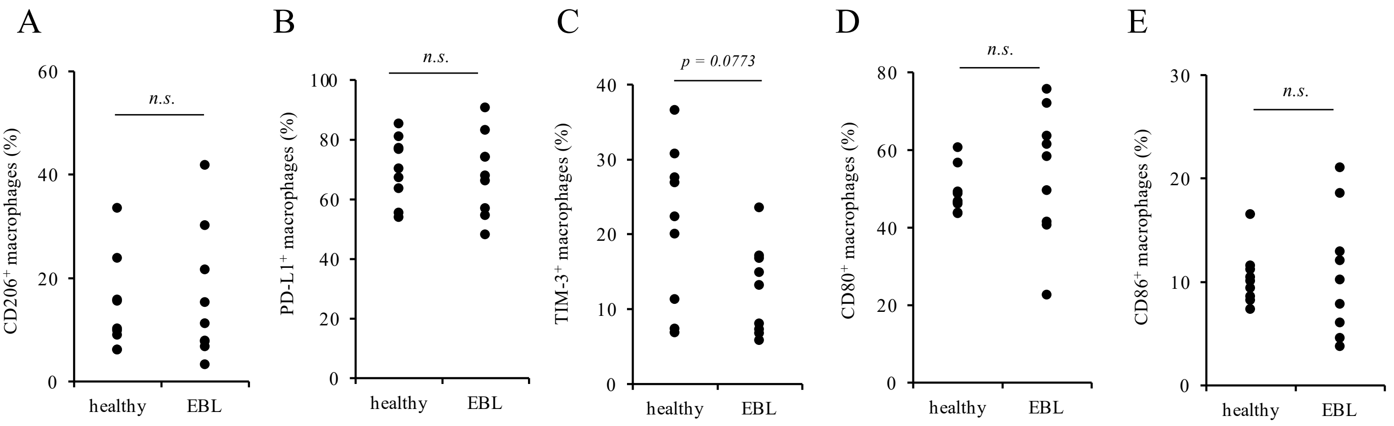


**Supplementary Figure 8. Phenotypic analysis of lymph node macrophages.**
Comparison of the proportion of macrophages expressing M2-macrophage related markers (A) $CD206^+$ cells, (B) PD-$L1^+$ cells, (C) TIM-$3^+$ cells, (D) $CD80^+$ cells, (E) $CD86^+$ cells. Data represent the proportion of marker-positive cells within the $CD11b^{hi}CD172a^+$ macrophage population. Data are shown for $n = 9$ animals per group. $^*P < 0.05$ (Mann–Whitney U test).

Supplementary Fig. 8

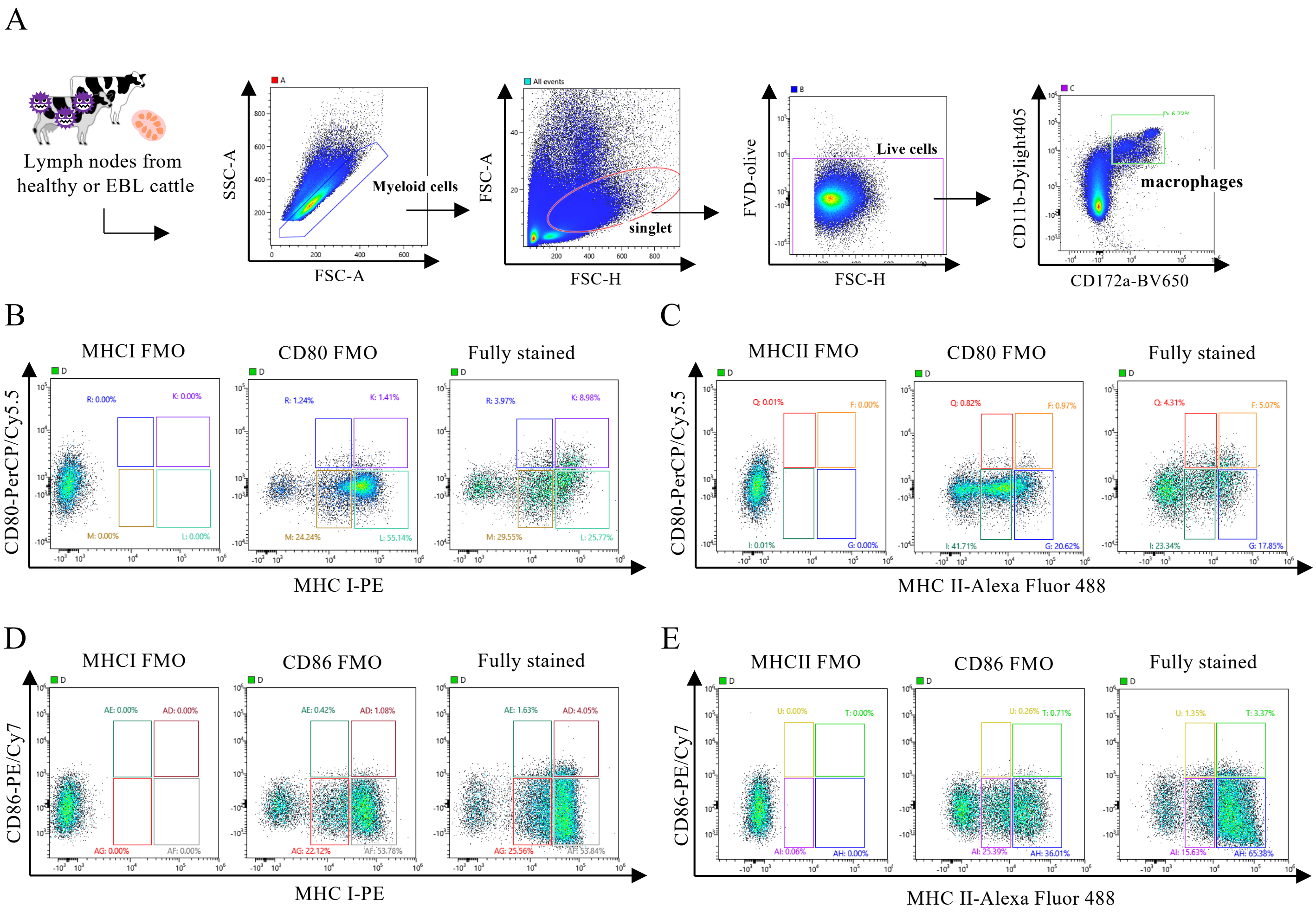


**Supplementary Figure 9. Gating strategy for analysis of MHC I/II and co-stimulatory molecules.** (A) Gating strategy used to define CD11b$^{hi}$CD172a$^{+}$ macrophage populations. (B-E) Representative flow cytometry plots within CD11b$^{hi}$CD172a$^{+}$ macrophages from lymph nodes (B) showing MHC I and CD80 co-expression, showing (C) MHC I and CD86 co-expression, (D) MHC II and CD80 co-expression, (E) MHC II and CD86 co-expression.

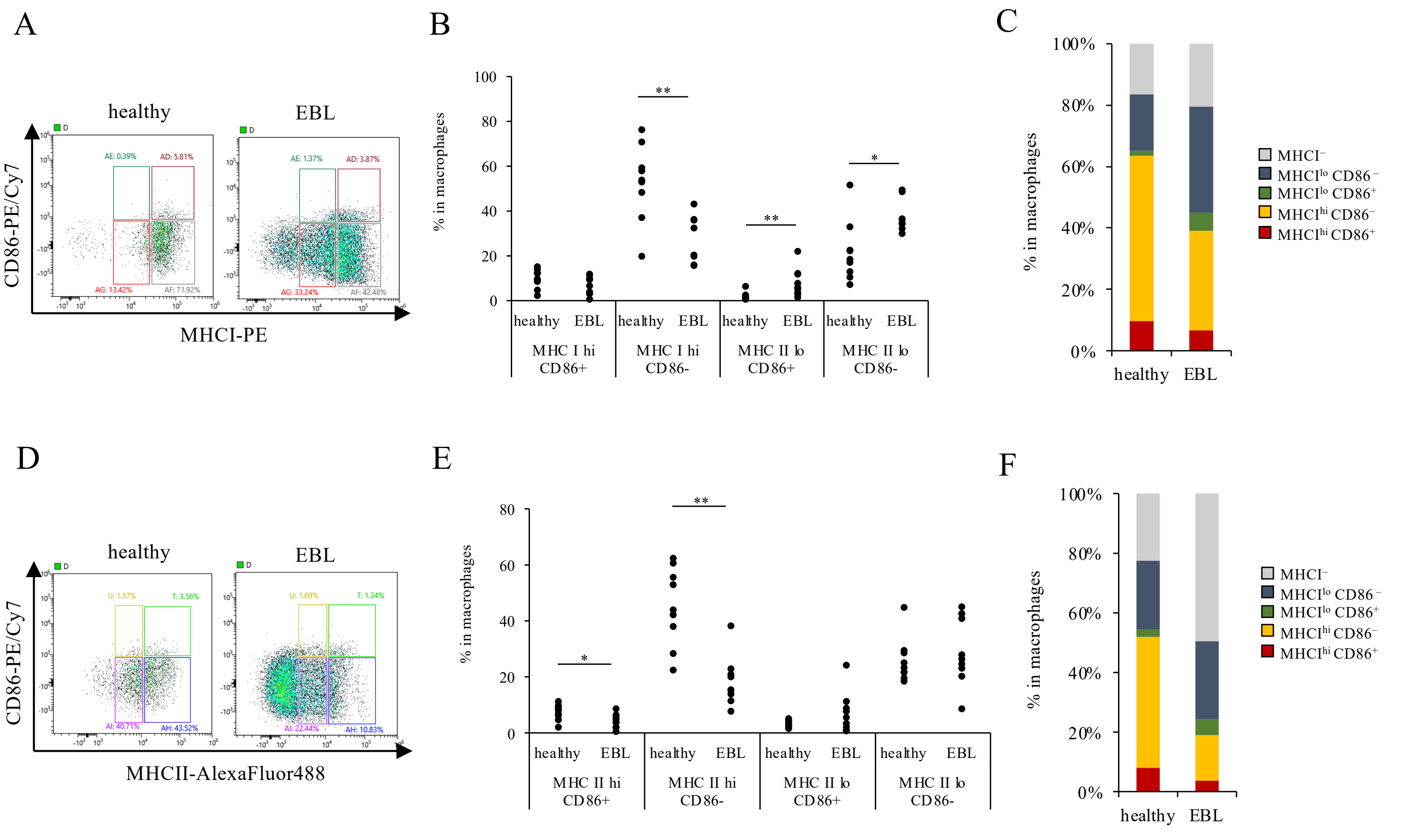


**Supplementary Figure 10. Altered co-expression of MHC molecules and CD86 in lymph node macrophages.**

(A) Distribution of macrophage subsets within the $CD11b^{hi}CD172a^{+}$ population in healthy and EBL tumor-bearing lymph nodes. (B) Representative plots showing the co-expression of MHC class I and CD86 in $CD11b^{hi}CD172a^{+}$ macrophages. (C) Frequencies of $CD86^{+}$ cells within MHC $I^{hi}$ and MHC $I^{lo}$ macrophage subsets in healthy and EBL lymph nodes. (D) Summary of the median distribution of CD86 co-expression within MHC $I^{hi}$ and MHC $I^{lo}$ macrophage subsets, displayed as a stacked bar graph. (E) Representative plots showing the co-expression of MHC class II and CD86 in $CD11b^{hi}CD172a^{+}$ macrophages. (F) Frequencies of $CD86^{+}$ cells within MHC $II^{hi}$ and MHC $II^{lo}$ macrophage subsets in healthy and EBL tumor-bearing lymph nodes. (G) Summary of the median distribution of CD86 co-expression within MHC $II^{hi}$ and MHC $II^{lo}$ macrophage subsets, displayed as a stacked bar graph. Data are shown for $n = 9$ animals per group. $*P < 0.05$ $**P < 0.01$ (Mann–Whitney U test).

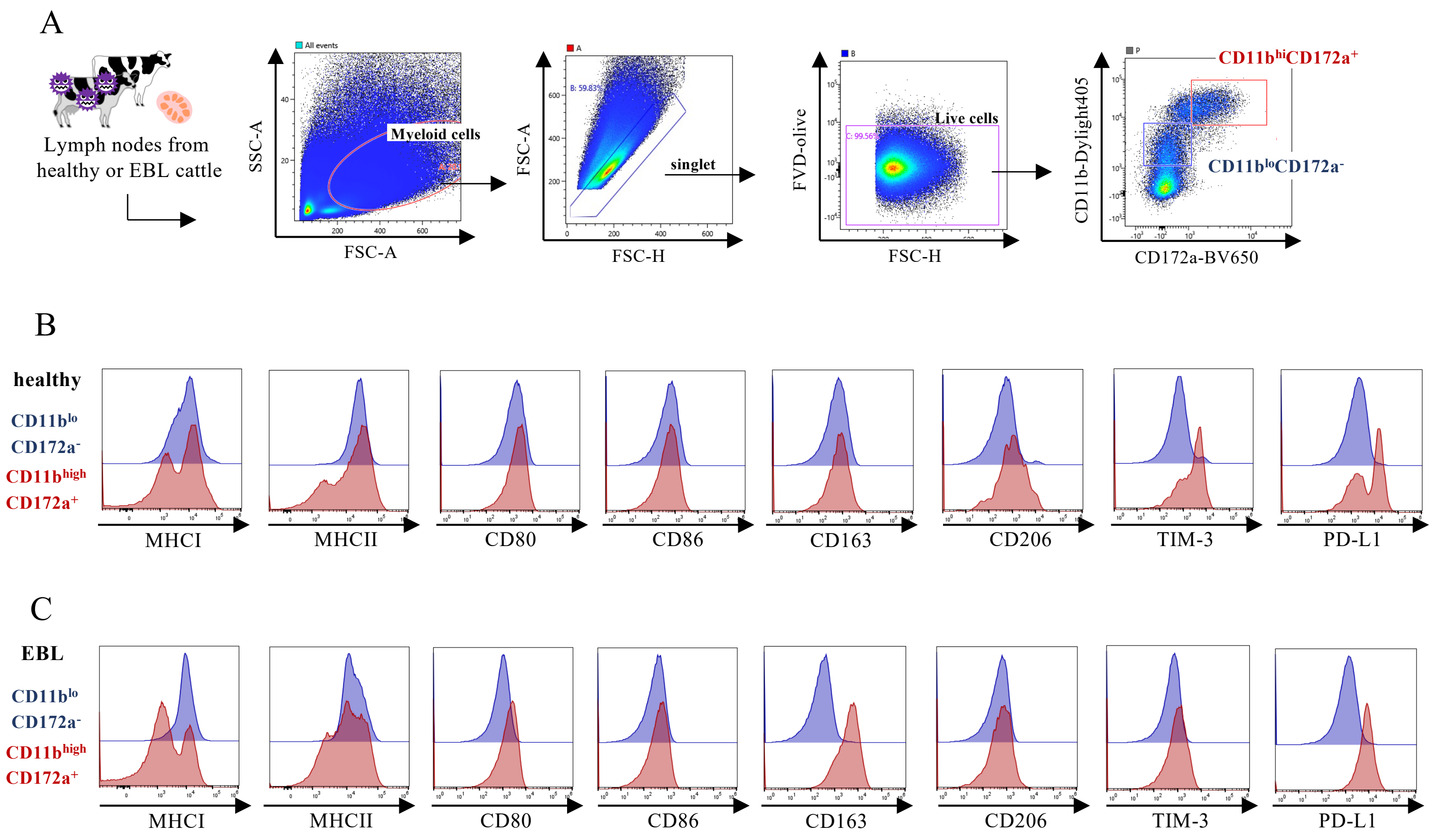


**Supplementary Figure 11. Comparative expression intensity of macrophage-associated markers between CD11b$^{hi}$CD172a$^{+}$ and CD11b$^{lo}$CD172a$^{-}$ cell populations.**

(A) Gating strategy used to define CD11b$^{hi}$CD172a$^{+}$ and CD11b$^{lo}$CD172a$^{-}$ populations for macrophage phenotyping. (B) Comparison of marker expression intensities between CD11b$^{hi}$CD172a$^{+}$ and CD11b$^{lo}$CD172a$^{-}$ populations in healthy lymph nodes. (C) Comparison of marker expression intensities between CD11b$^{hi}$CD172a$^{+}$ and CD11b$^{lo}$CD172a$^{-}$ populations in EBL tumor lymph nodes.
